# Self-Assembled, Hierarchical Structured Surfaces for Applications in (Super)hydrophobic Antiviral Coatings

**DOI:** 10.1021/acs.langmuir.2c01579

**Published:** 2022-08-17

**Authors:** Frances Dawson, Wen C. Yew, Bethany Orme, Christopher Markwell, Rodrigo Ledesma-Aguilar, Justin J. Perry, Ian M. Shortman, Darren Smith, Hamdi Torun, Gary Wells, Matthew G. Unthank

**Affiliations:** # Northumbria University, Newcastle upon Tyne NE1 8ST, U.K.; § Defence Science and Technology Laboratory, Porton Down, Salisbury SP4 0JQ, U.K.; ∇ Institute for Multiscale Thermofluids (IMT), School of Engineering, University of Edinburgh, Mayfield Road, Edinburgh EH9 3JL, Scotland, U.K.

## Abstract

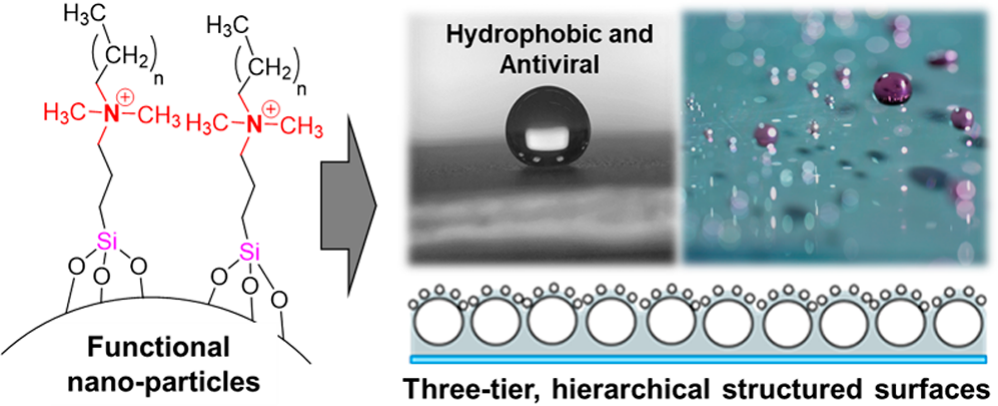

A versatile method for the creation of multitier hierarchical structured surfaces is reported, which optimizes both antiviral and hydrophobic (easy-clean) properties. The methodology exploits the availability of surface-active chemical groups while also manipulating both the surface micro- and nanostructure to control the way the surface coating interacts with virus particles within a liquid droplet. This methodology has significant advantages over single-tier structured surfaces, including the ability to overcome the droplet-pinning effect and in delivering surfaces with high static contact angles (>130°) and good antiviral efficacy (log kill >2). In addition, the methodology highlights a valuable approach for the creation of mechanically robust, nanostructured surfaces which can be prepared by spray application using nonspecialized equipment.

## Introduction

The creation of biomimetic structured surfaces with embedded reactive surface functionality offers an opportunity to precisely control the way that objects and surfaces interact with their environment.^1−3^ Controlling the way that liquids, gases, pollutants, microorganisms, viruses, and other abundant environmental materials interact with a structured surface can allow tailoring of surfaces to deliver multiple complementary properties through careful, informed design. The COVID-19 pandemic^4^ has raised the awareness and requirement for technologies that can combat the spread of pathogenic viruses.^5−8^ In the field of surface chemistry, two particular topics that were widely discussed were (i) the use of antiviral surface coating to deactivate pathogens on contact^9,10^ and (ii) the ability to rapidly decontaminate infected areas, including hospitals, ambulances,^11^ equipment, and public areas.

While mechanically robust surfaces that deliver both long-term antiviral and self-cleaning properties are desirable, the creation of such systems is fraught with technical challenges. Self-cleaning, superhydrophobic surfaces that also incorporate chemically active antimicrobial/antiviral properties are difficult to engineer.^5,12^ Alternatively, more hydrophilic metal oxide coatings^13^ make less effective “self-cleaning” surfaces^14^ or require a controlled depletion or “polishing” mechanism in order to keep the surface free from fouling.^15^ The use of tethered virucidal functionality is essential for a low-maintenance, environmentally compliant approach to long lasting antiviral efficacy, but this approach comes with conflicting surface properties. Balancing a strategy to repel attachment while delivering reactive chemical biocidal efficacy is a well-known conundrum being investigated to counter surface fouling.^16,17^ Methodologies exist to create superhydrophobic coatings by spray application of nanoparticle suspensions,^18^ but to our knowledge this approach has not been combined with organic chemical functionality designed for contact antiviral surface efficacy to deliver robust structured coatings. Equally limiting, even commercially available coatings based on nanoparticle suspensions (Glaco Mirror Coat Zero from Soft99 Co)^19^ suffer from extremely poor mechanical and abrasion resistance, limiting their lifetime and use case. User-friendly and robust structured surfaces are less commonly described,^20,21^ with most requiring complex or scale-limiting application processes,^22,23^ rendering them less suitable for wide-scale adoption.

In this paper, we present a new self-assembled multitiered, hierarchical coating system that delivers both high degrees of water repellence and antiviral efficacy. In addition to this, we describe a scalable strategy for the creation of mechanically robust, multitiered structured surfaces that can tolerate numerous abrasion cycles while maintaining their functional surface properties. Using an established bacteriophage (Phi6)^24^ as a surrogate model for the SARS-CoV-2 virus^25,26^ in droplets,^27−29^ we report relevant antiviral properties that are driven by both micro/nano-structure and surface chemistry.

## Experimental Section

### Materials

The OH-functional (hydroxyl-functionalized) silica particles used were AEROSIL300 (7 nm diameter), AEROSIL90 (20 nm diameter), AEROSILOX50 (40 nm diameter), and SIPERNAT 350 (4500 nm/4.5 μm diameter) supplied by Evonik Industries. Silane functionalization agents *n*-tetradecyldimethyl(3-trimethoxysilylpropyl)-ammonium chloride (C14QUATSi, 50% in methanol), dimethyloctadecyl[3-(trimethoxysilyl)propyl]ammonium chloride (C18QUATSi, 60% in methanol), trimethoxy(octadecyl)silane (C18Si), and *N*-trimethoxysilylpropyl-*N*,*N*,*N*-trimethylammonium chloride (QUATSi, 50% in methanol) were supplied by FluoroChem or Fisher Scientific and used as received. All solvents used were standard laboratory grade supplied by Fisher Scientific. Infrared analysis was carried out using a Bruker Platinum-ATR and OPUS 7.0 software.

### Particle Functionalization and Formulation

OH-functional silica particles (0.75 g) were suspended in either an anhydrous or hydrous environment: toluene (75 mL, ANHYD) or ethanol:water (50:50, 75 mL, HYD), respectively. Following literature procedures,^30,31^ each suspension was treated with silane functionalization agent (0.75 mmol) and heated for 4 h at reflux. Suspensions were allowed to cool before centrifuge sedimentation (3000 rpm, 10 min), decanting the solvent, and washing three times with ethanol and once with propan-2-ol (centrifuging and decanting in between each washing cycle). The silicas (0.75 g) were then suspended in propan-2-ol (75 mL), and sonication was applied with a MSE Soniprep 150 Plus tip sonicator (15 min, 14 μm amplitude, over ice) to produce a stable silica suspension for spray application to glass substrates.

### Coating Application

Glass microscope coverslips were adhered to microscope slides (3 per slide) with pressure adhesive putty. All samples were then spray-coated with silica suspensions (1 wt % in propan-2-ol, 5 coats) using a Sparmax spray gun (GP-35) fitted with a hopper and air compressor (Fengda FD-196 Piston Type 186W) allowing 20–30 s for the substrates to dry between coats. The coverslips (still attached to the glass microscope slides) were then immersed in distilled water for 5 min, removed, and rinsed with 50 mL of distilled water from a measuring cylinder. Excess water was shaken off, and the samples were allowed to dry at room temperature overnight. For mechanically robust structured surfaces, microscope slide were treated directly with a solution of PDMS (Silgard 184 base and curing agent), with platinum-divinyltetramethyldisiloxane in hexane, optionally containing C18Si microsilica (4.5 μm, hierarchical only). Abrasion testing and contact angle analysis were conducted on the coated microscope slides (see Supporting Information for full details).

### Antiviral Testing

Using a modified method derived from Haldar et al.,^32^ to allow semihigh throughput testing, a high titer stock of bacteriophage Phi6 (DSM 21518) was raised against bacteria *Pseudomonas syringae* (DSMZ 21482) (kindly provided to DSMZ by Sylvain Moineau, University of Laval, Quebec, Canada).^33^ Bacteriophage titers of >1 × 10^8^ pfu.ml were used as part of the surface tests. Each coverslip (test surface or glass control) were placed into individual wells on a 6-well plate. To each test surface 10 μL of bacteriophage stock in Lysogeny broth (LB media and 0.01 M CaCl_2_) was added to the surface and immobilized across the slide by addition of a coverslip. This was then incubated for 1 h at room temperature (∼18–20 °C). The samples were submerged in 1 mL of LB buffer, the coverslip was removed, and surfaces were washed with gentle pipetting. The test LB containing remnant bacteriophages were subject to 10-fold serial dilution. A phage overlay plate was created by the lower layer LB buffer with 1.2% *w/v* Difco Bacterial Agar, overlaid with LB with 0.4% *w/v* Difco agar, containing 100 μL of *Pseudomonas syringae* with culture optical density of 0.5–0.7 OD600. To the bacterial overlay, 10 μL of each dilution was added, and each sample was allowed to dry and incubated ∼18 h at 25 °C. Individual plaques were counted to offer the remaining viable bacteriophages after testing.

### Static and Dynamic Contact Angles

Static contact angles were all measured using a Krüss drop shape analyzer (DSA30). For each sample, 4 × 2 μL droplets were measured, and an average contact angle with standard deviation was recorded using the built-in angle tool on the DSA. To measure the contact angle hysteresis (CAH), samples were placed onto an Aerotech 2 axis tilting stage mounted on Thorlabs XYZ translational stages and leveled with a Level Developments Engineering level accurate to 50 μm in the meter. Droplets were generated using an Exigo microfluidic syringe pump, held above the surface using a Thorlabs translational stage. The initial DI water droplet volume was 2 μL, generated at 0.5 μL/s, through a 0.7 mm outer diameter flat-tipped needle before being placed onto the surface. To identify the surfaces advancing angle, the original 2 μL droplet was inflated by 4 μL at a flow rate of 0.1 μL/s. The flow was then reversed (−0.1 μL/s) to deflate the droplet and obtain the receding angle. In some cases, the removal of 4 μL of DI water in the deflation step did not result in motion of the pinned contact line; therefore, the initial 2 μL of liquid was removed as well. The inflation and deflation procedures were recorded with a Navitar 4.0× zoom lens and 0.5× objective attached to an IDS USB camera. The subsequent advancing and receding angles were extracted from the corresponding frames of the video using the angle tool in ImageJ.^34^ Five droplets per sample type were measured to get average receding/advancing angles.

### Atomic Force Microscopy

The topographic images of the coating surface were acquired using a commercial AFM system (Veeco DI3100, Bruker Corporation). Tapping mode imaging techniques were applied using a cantilever with a stiffness of 26 N/m and tip radius of 7 nm (OTESPA, Bruker). The samples were imaged with different scan sizes to investigate the hierarchical structures.

## Results and Discussion

Cationic quaternary ammonium groups coupled with long (C8–C18) alkyl chain functionality are known to deactivate both viruses and bacteria through disruption of their lipid membrane envelopes.^35−37^ In addition, the functionalization of surfaces with these moieties have been shown to retain antimicrobial and antiviral properties.^38−40^ Silica nano- and microparticles were accordingly functionalized with a range of alkoxysilanes containing (i) cationic C14/C18 alkyl quaternary ammonium groups (C14QUATSi and C18QUATSi), (ii) neutral C18 alkyl group (C18Si, control), and (iii) cationic trimethyl quaternary ammonium group (QUATSi, control) as outlined in Table 1. The C18Si and QUATSi agents were included in the study as controls, which lacked either (i) the cationic functionality (C18Si) or (ii) the hydrophobic alkyl chain (QUATSi) required for optimal antiviral efficacy.^41,42^

**Table 1:**
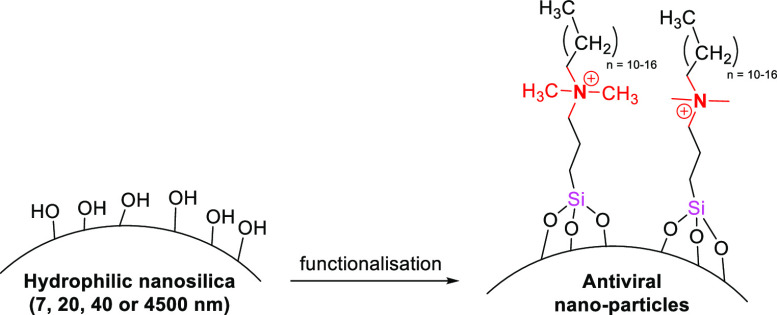

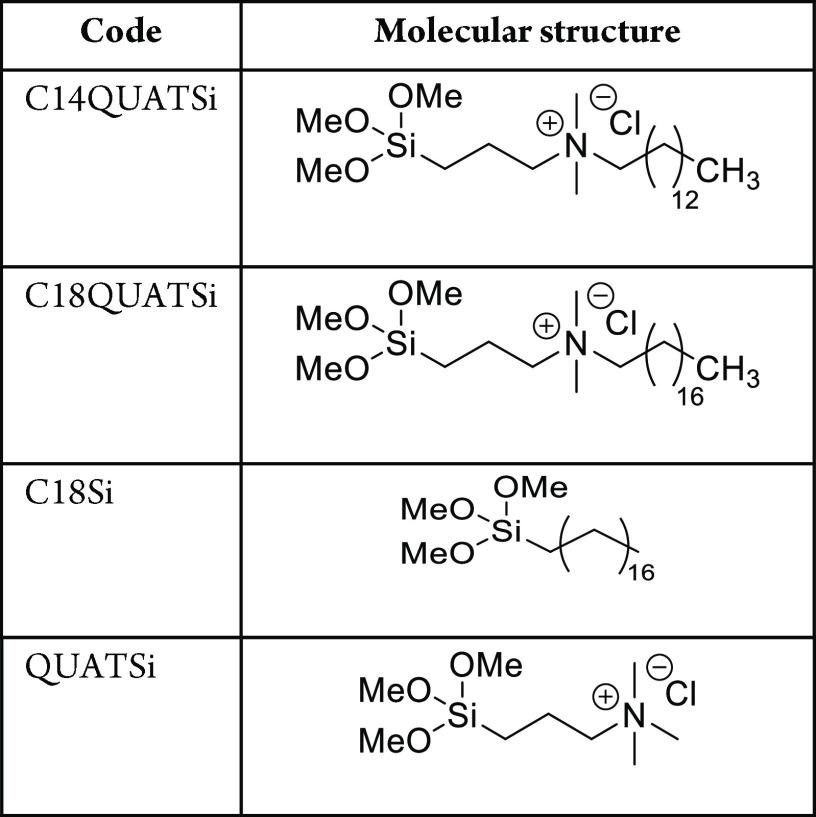
Structure of Silane Functionalization Agents Used in the Preparation of Surface Functional Nano- and Microsilica Particles

A range of hydrophilic (Si–OH functional) nano- and microsilica particles (commercial grade, Evonik Ind. AG, [7, 20, 40, and 4500 nm diameter]), were surface functionalized using a modification of two established methodologies,^30,31^ one hydrous method (HYD, ethanol–water, reflux, 4 h) and one anhydrous method (ANHYD, toluene, reflux, 4h). Using these two functionalization methodologies with the four functional silanes (Table 1) and four different diameter silicas gave a related set of materials for study. Successful functionalization of silica particles was confirmed by infrared spectroscopy which showed C–H stretching (3000–2840 cm^–1^) and C–H bending (1465 cm^–1^), characteristic of the alkyl functional silanes (see the Supporting Information for IR spectra).

**Figure 1:**
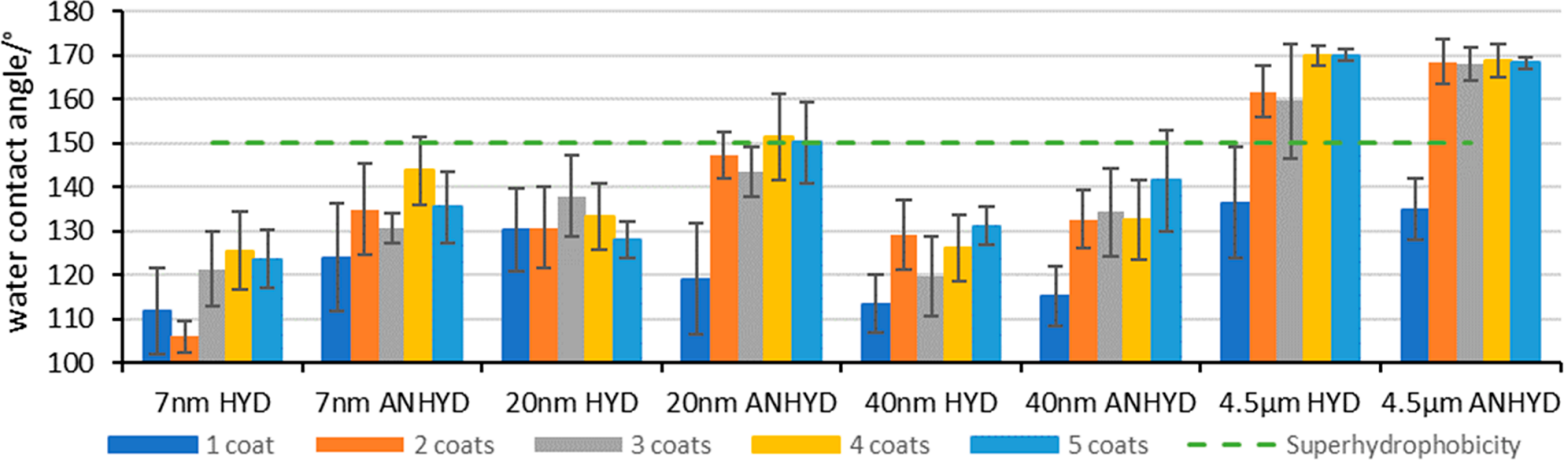
Effect of number of coats and functionalization method on average water contact angle of spray-coated slides across a range of different particle sized silicas, functionalized with the hydrophobic C18Si agent (Table 1).

To understand the impact of surface coverage on static contact angle, C18Si (hydrophobic) silica suspension covering the 7, 20, 40, and 4500 nm diameter range was spray applied onto glass microscope slides (1 wt % in pronaol-2-ol) with increasing numbers of coats (from 1 to 5 coats), and the static water contact angle was measured in triplicate on each coating (*n* = 3). A single spray coat appeared to deliver incomplete coverage of the substrate in certain cases, which was most significant in the 4.5 μm and 40 nm examples (HYD and ANHYD, see Figure 1) as evidenced by further increase in static contact angle on application of additional coats (i.e., 2–5 coats).

Some general trends in static contact angle could also be observed when comparing the surface functionalization methods, particularly in the lower particle size range. Within the 7 nm series, the “anhydrous method” (ANHYD) appears to deliver more hydrophobic surface properties, independent of number of coats, as indicated by generally higher static contact angles in this series (Figure 1, 7 nm, HYD vs ANHYD examples). This data set indicates that superhydrophobicity (static contact angle >150°)^43,44^ is achievable through control of particle size, with the larger 4500 nm examples (4.5 μm, treated with hydrophobic C18Si) consistently delivering contact angles above 150°, while the nanostructured surfaces were generally <150° and therefore not indicative of a superhydrophobic surface.

**Figure 2:**
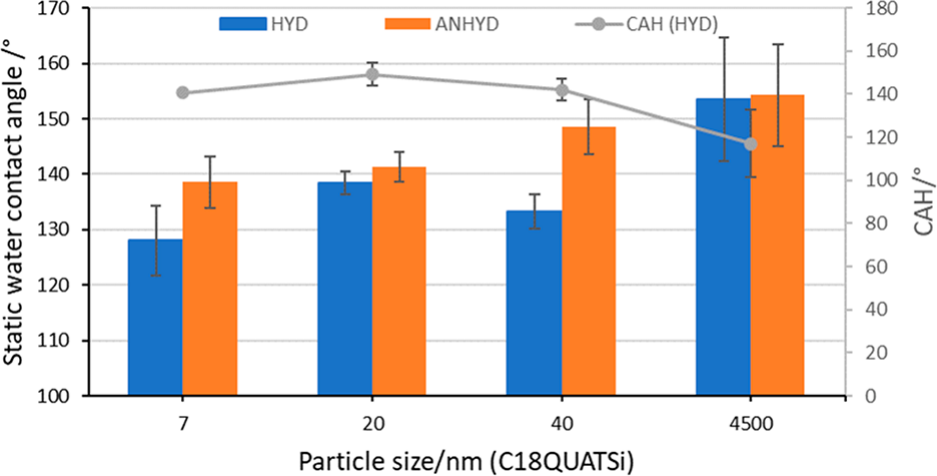
Effect of particle size on static water contact angle and CAH functionalized with C18QUATSi under hydrous (blue) and anhydrous (orange) conditions (5 coats). CAH shown for anhydrous series.

Structured surfaces based on the hydrophobic quaternary ammonium (antiviral) functionalization agent C18QUATSi showed a gradual general increase in static water contact angle with particle diameter (Figure 2). Functionalized under both hydrous (blue) and anhydrous (orange) methods, the contact angles increased to ∼154° for 4.5 μm from ∼128°/138 (HYD/ANHYD) for 7 nm. These data are in general lower than the equivalent surfaces functionalized with the C18Si functionalization agent, which can achieve a static contact angle of >160°.

A superhydrophobic surface can be characterized as having both a high static contact angle and a low contact angle hysteresis (CAH) (e.g., a high receding angle).^43,44^ The advancing and receding angles for all structured surfaces were measured, giving a measurement of CAH. Figure 2 (right *y*-axis) shows CAH for C18QUATSi (HYD) structured surfaces across a range of different particle sizes. All entries show very high CAH (117–149°) regardless of particle size across the C18QUATSi series. The high degrees of CAH are linked to the presence of the polar quaternary ammonium group embedded within the nonpolar alkyl chain surface functionality, leading to amphiphilic surface characteristics of high static contact angle but high CAH (due to water droplet pinning). This is supported by a much lower CAH (28°, Supporting Information) for an equivalent (40 nm diameter) structured surface functionalized with the C18Si functionality (i.e., lacking the amphiphilic character of C18QUATSi).

**Figure 3:**
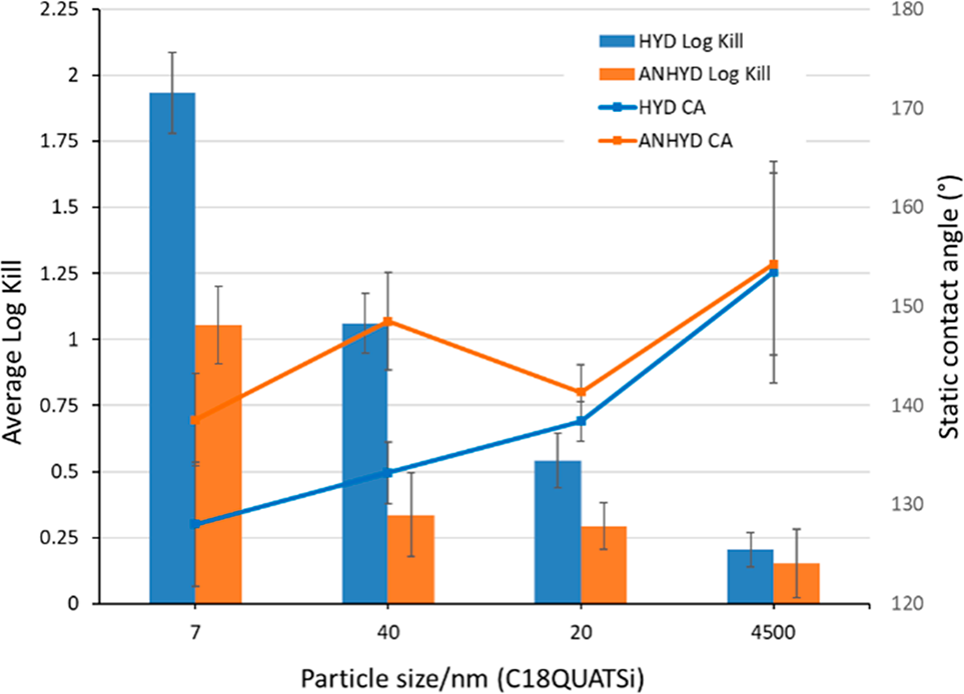
Relationship between antiviral efficacy and static contact angle in C18QUATSi functional coatings, across a range of particle sizes.

Antiviral testing of the full range of surfaces was conducted to understand the relationship between antiviral efficacy and hydrophobicity. This was performed using the bacteriophage Phi6, an enveloped surrogate model for the SARS-CoV-2 virus^24−26^ in a semi high through-put method developed for testing spray coatings for antiviral surface activity.^32^ This bacteriophage was selected as its morphology resembles SARS-CoV-2 in that it is a similar size, is encapsulated within a lipid layer, and possesses surface spikes and an RNA genome. Phi6 has been used in previous studies as a surrogate for respiratory viruses, including influenza virus H5N1.^25^ Accordingly, 20 mm × 20 mm glass slide covers were coated (in triplicate) and immersed in 6-well plates. Bacteriophage containing droplets were incubated for 1 h on these coated surfaces, and the reduction in viral titer was measured using established methodologies (see the Supporting Information for details).^32^ Across the full range of coatings, an inverse correlation was generally identified between antiviral efficacy (measured by log kill) and hydrophobicity. Figure 3 shows a representative subset of the data to illustrate this trend with C18QUATSi functional particles.

In the most efficacious example (C18QUATSi, HYD, 7 nm) the “single-tiered” (i.e., single particle size used) structured coating achieved a log kill of 1.93 with a corresponding static contact angle of 128°. Across the 7–40 nm particle sizes, the hydrous functionalization method (“HYD”, Figure 3, blue) shows both higher log kill and corresponding lower static contact angles in comparison to the “anhydrous” functionalization method (“ANHYD”, Figure 3, orange). As expected, the C18Si functional coating (no quaternary ammonium functional group) delivered very high static contact angle (∼160°) but no detectable antiviral efficacy (i.e., zero log kill). In contrast, the QUATSi (no hydrophobic alkyl chain) delivered the highest log kill values of 2.5–3.0, with complete surface wetting (i.e., no measurable static contact angle). This highlights the importance of surface contact angle on antiviral action in functional coatings, where the antiviral efficacy of the resulting coating is related to contact area of the water droplet containing virus particles. Higher contact angle implies less of the water droplet (and its viral load) is in contact with the surface at any given time. In single-tiered structured coatings, a balance between hydrophobicity and antiviral efficacy is required to deliver effective antiviral surfaces that also incorporate a degree of easy-clean/hydrophobic behavior. To explore this relationship further, the degree of hydrophobicity was tuned using a mixture of hydrophobic C18QUATSi (alkylated) with hydrophilic QUATSi (nonalkylated) functionalities across a single particle size, thus mapping the structure–property relationship of “single-tiered” structured coating.

**Figure 4:**
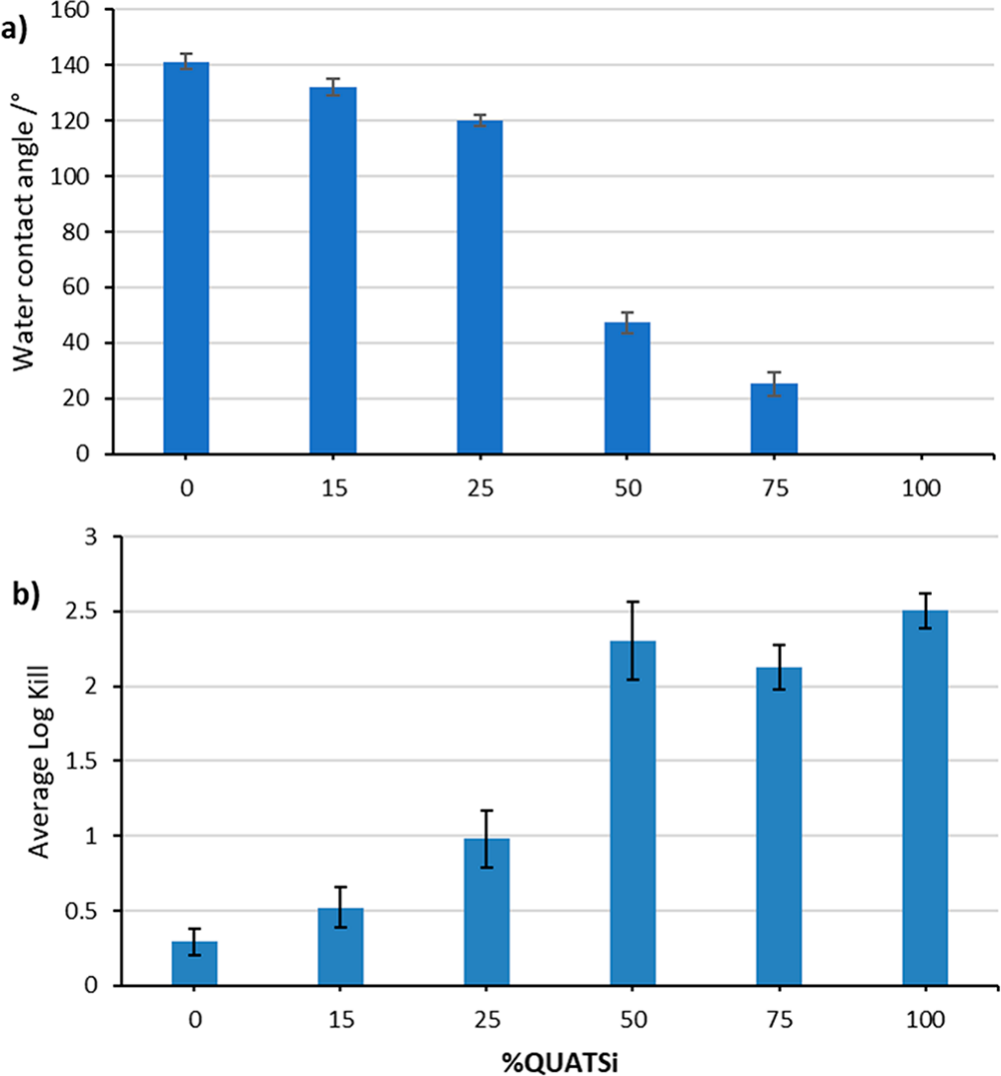
Control of hydrophobicity and antiviral efficacy in nanostructured coatings (20 nm) via a mixed functionality approach using QUATSi (hydrophilic) and C19QUATSi (hydrophobic) functionalization agents (*x*-axis represents mole% of QUATSi, with the remainder as C18QUATSi)

A series of functionalized silica particles with varying degrees of hydrophobicity were produced from a single sized silica particle (20 nm) using a mixture of C18QUATSi (hydrophobic, 0–100%) and QUATSi (hydrophilic, 100–0%) in varying molar proportions, according to Figure 4. The static water contact angle data and log virus kill are shown in Figure 4 and again demonstrate the previously determined trend; as the surface becomes more hydrophilic, the antiviral efficacy increases. This data strongly supports the hypothesis that the ability of the functional coating to inactivate the virus is related to contact angle/contact area^45^ of the water droplet containing the virus in this type of single-tier structured coating.

Specifically, there is a large increase in antiviral log kill (to 2.1–2.3 from less than 1) when 50 mol % QUATSi (hydrophilic) is used with 50 mol % C18QUATSi (hydrophobic) (see Figure 4, entry 4), which corresponds to a significant drop in the contact angle to <47°. At contact angles below 47°, the log kill values plateau and no further benefit in antiviral efficacy is realized. This study shows for the first time that (at a fixed particle size) the molecular surface structure can be tuned using silica-particle functionalization agents to produce tunable surface properties for antiviral coatings. This study also demonstrates the limitations of this approach when trying to achieve a surface with both a very high contact angle (i.e., > 130°) that also exhibits high log kill values (i.e., above 1.5–2.0). One additional, yet significant drawback with this approach was water droplet pinning and the resulting high CAH of (51–147°), across the series of surfaces, compromising the ability of a coating to deliver easy-clean properties.

### Self-Assembled Hierarchical Structured Surfaces

**Figure 5:**
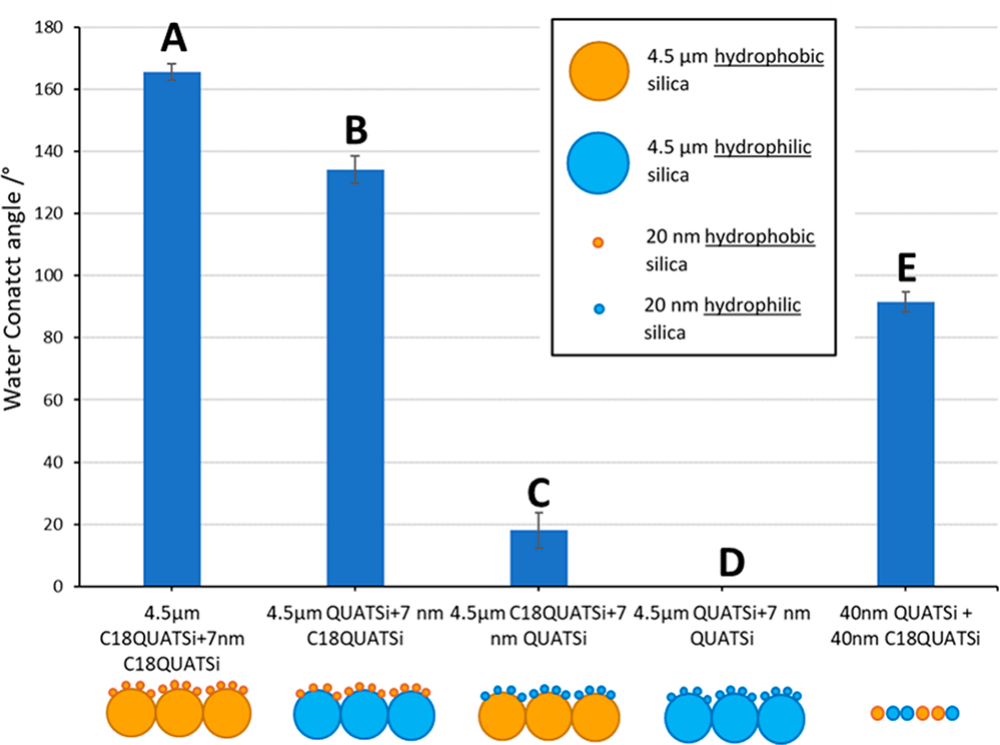
Static water contact of hierarchical structured surfaces based on 4.5 μm and 7 nm functionalized (C18QUATSi and C18Si) silica particles, prepared by a single spray application (also see Figure 7).

Hierarchical structured surfaces deliver superhydrophobicity by a different mechanisms which may avoid the negative pinning characteristics of the single-tiered coatings.^46^ Biomimetic hierarchical systems, inspired by the lotus leaf effect,^3,47,48^ could represent a step-change in performance and deliver new insight into the structural and molecular design criteria for functional, multitiered, structured surfaces. A range of self-assembled, hierarchical structured surfaces were prepared using a bimodal silica suspension mixture, prepared with both the hydrophobic (C18QUATSi) and hydrophilic (QUATSi) functionalized antiviral nano/micro-particles. The resulting suspensions were spray applied to glass substrates to create a range of hierarchical structured coatings with unique properties (Figure 5). Their wetting behavior, antiviral efficacy, and surface structure were then studied.

Structured coating system **A** (nano- and microparticles functionalized with C18QUATSi) showed the highest static contact angle seen thus far from amphiphilic C18QUATSi functional particles (>165°, Figure 5, entry 1). This is significantly beyond that possible from single-tiered nano- or microstructured surfaces (i.e., 7 nm particles give a contact angle of only 139° and 4.5 um particles give a contact angle of 154°). Most importantly coating system **A** demonstrated a step-change in CAH, measuring only 7° (in comparison to 140–142° for the individual nano- and microstructure surfaces) effectively eliminating the water drop pinning behavior previously seen. Using this approach to control contact angle behavior, the nature of surface chemical functionality has been decoupled from the wetting behavior. Specifically the hierarchical structured surface overrides the amphiphilic “droplet pinning” nature of C18QUATSi-based coatings (both the nano- and microsized) delivering both high static contact angles with low CAH. With this chemical treatment, water droplets could be observed rolling across a surface to deliver easy-clean or even self-cleaning performance characteristics from a superhydrophobic surface (see the Supporting Information for a video of droplet behavior).

To probe this effect further and to demonstrate this technique’s ability to control surface and wetting behavior, we explored the properties of coating systems B–E (Figure 5). Structured coating B was created with 50 wt % hydrophilic (QUATSi) microparticles and 50 wt % hydrophobic (C18QUATSi) nanoparticles. These particles self-assembled into a structured surface with the C18QUATSi antiviral hydrophobic nanoparticles layer over a larger dimension QUATSi microparticle base layer. Experimentation showed that the surface properties of this coating are dominated by the hydrophobic nature of the nanostructured upper tier, delivering a high static contact angle of 134°. The exposed surface of the coating is therefore composed of hydrophobic (C18QUATSi) nanoparticles rather than hydrophilic (QUATSi) microparticles. This indicates that the bimodal suspension of particles **self-assemble on application** to deliver a hierarchical structured surface. By contrast, the relative hydrophobicity of a nonhierarchical structured surface based on the same wt % composition of hydrophilic:hydrophobic particles (both 40 μm) is substantially lower than the hierarchical equivalent (Figure 5, entry B (134°) vs E (92°)). Entry C, Figure 5, shows that the surface properties of the hydrophobic microparticles (4.5 μm, C18QUATSi) are masked by the hydrophilic nanoparticles (20 nm, QUATSi), exhibiting a hydrophilic surface dominated by the properties of the nanoparticles with very low contact angle (18°). Entry D composed of hydrophilic nano- and microparticles (QUATSi) delivers a fully wettable surface (no visible droplets) due to complete surface wetting.

**Figure 6:**
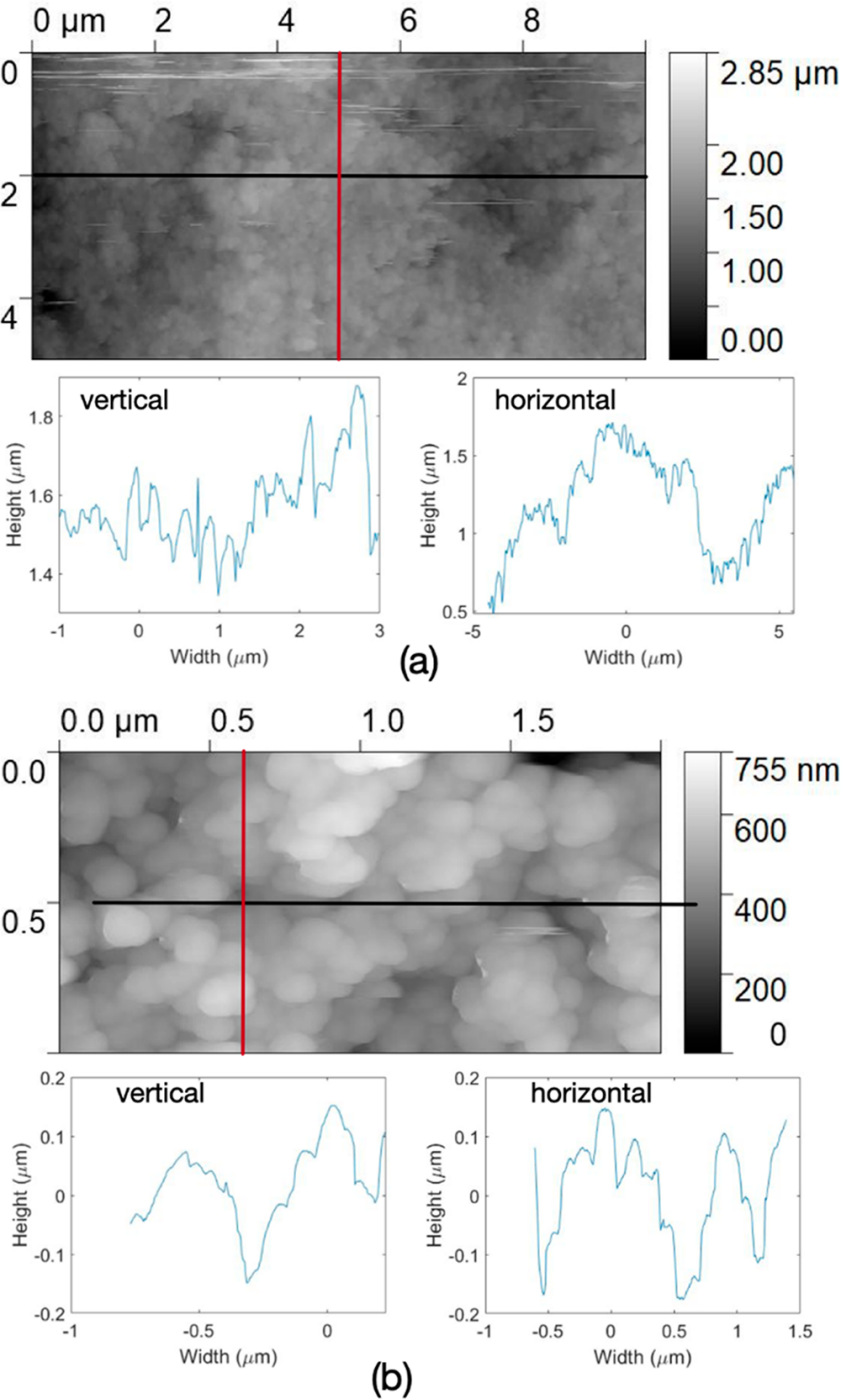
Topographic images acquired using AFM over an area of (a) 10 × 5 μm and (b) 2 × 1 μm, showing the presence of a hierarchical nano- on microsurface structure. Horizontal and vertical line scans are obtained over the red and black lines shown in the topographic images.

The hierarchical structure of these self-assembled surfaces was studied and confirmed by atomic force microscopy (AFM) across a range of scan dimensions. Figure 6a shows an image over an area of 10 × 5 μm, where the presence of 4.5 μm particles over the surface results in the larger topographic differences, while the higher-frequency variations confirm the presence of nanoparticles across the structured surface. The presence of 7 nm nanoparticles is clearly visible when the scan area is smaller, as shown in Figure 6b. Statistical analysis of different samples is provided in the Supporting Information.

**Figure 7:**
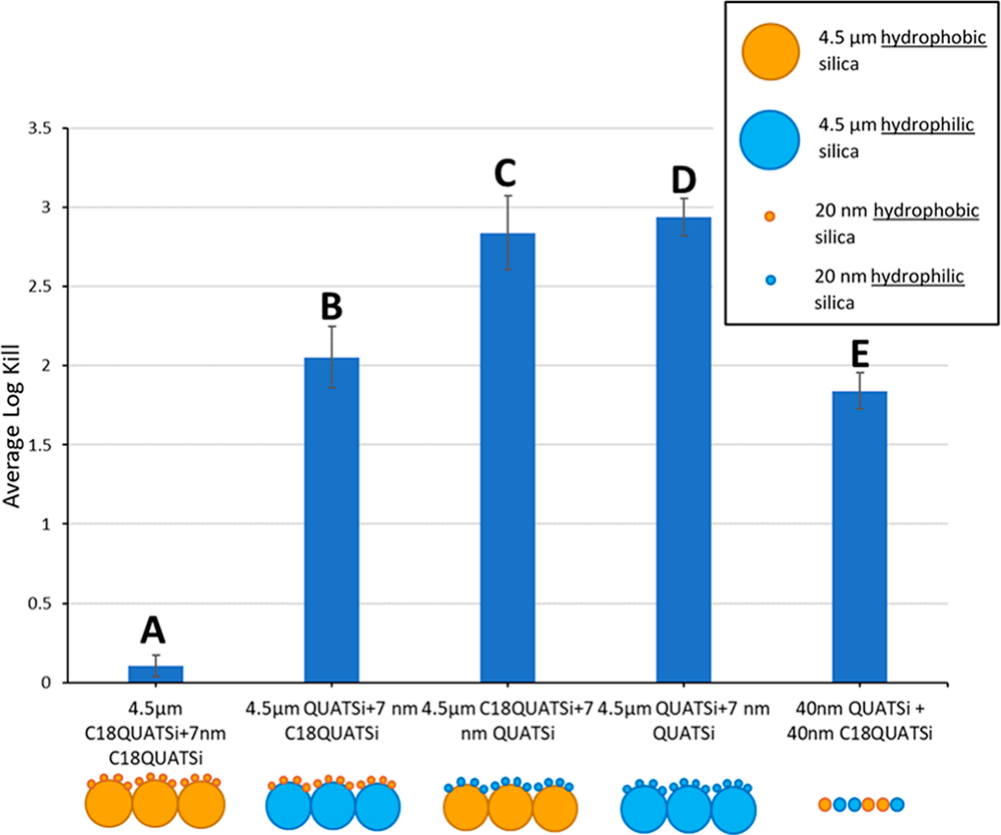
Antiviral efficacy evaluation of self-assembled hierarchical structured surfaces from 4.5 μm and 7 nm functionalized (C18QUATSi and C18Si) particles, prepared by a single spray application.

The hierarchical structured coatings also exhibited greater antiviral efficacy, in comparison to nonhierarchical surfaces (Figure 7). Structured surface B in-particular (hydrophilic microstructured base layer with a nanostructured antiviral, hydrophobic surface) exhibited both high water repellence (contact angle = 134°, Figure 5, entry B) and high antiviral efficacy (log kill of >2, Figure 7, entry B). This is a significant improvement over log kill values of only 0.3–0.5 for similar contact angles, 132–141°, for the equivalent nonhierarchical coating (as shown in Figure 4). Hydrophilic, hierarchical coatings based on nanostructured antiviral hydrophilic particles structured over either a hydrophobic (Figure 7, entry C) or hydrophilic (Figure 7, entry D) microstructure also showed higher log kill (2.8–2.9) in comparison to nonhierarchical surfaces of equivalent static contact angle (Figure 4, 50–100% QUATSi, log kill 2.1–2.5). As expected, structured surface A exhibited relatively low antiviral efficacy, presumably due to the exceptionally high static contact angle >165° (entry A, Figure 7).

While hydrophilic examples (Entries C and D, Figure 5 and Figure 7) deliver optimal antiviral efficacy, this study shows that a combination of both high static contact angle (i.e., hydrophobicity) and high antiviral efficacy (observed in entry B, Figure 5 and Figure 7), can be achieved via a “multitiered” hierarchical surface structure. The “multitiered” structure, promotes the water droplets into a nonwetting (Cassie–Baxter) state,^43,44^ while the high surface area, resulting from the nano-on-micro hierarchy, ensures a high concentration of antiviral alkyl quaternary ammonium groups are present at the surface (see Supporting Information, Figure S7a and S7b). This synergist combination of effects delivers both high degrees of hydrophobicity and good antiviral efficacy.

### Robust Hydrophobic Surfaces

A major challenge in the use of micro- and nanostructured coatings for general applications, is overcoming mechanical robustness issue.^19−21^ Mild abrasion or even skin contact is often enough to significantly deteriorate the hydrophobic properties of such coatings.^20^ This phenomena also proved to be true in the hierarchical structured surface described above. We therefore developed a new synthetic methodology to create hierarchical structured coatings, that are both mechanically robust and allow chemically active functional groups to assemble at the coating surface. A range of polymeric binders/adhesives were studied to find a suitable system that could (i) form a thin, continuous polymer layer on glass; (ii) was optically transparent and colorless; and (iii) could adhere to our selected substrates. A formulation based on a 2-component polydimethylsiloxane (PDMS, Sylgard 184) with additional Karstedt catalyst in hexane was found to deliver the required properties. To successfully adhere a structured silica-layer to a substrate using this approach, the binder must be first applied to the substrate followed by a suspension of silica in a two-coat application process. For optimal application of the second (silica) coat, the binder (1st coat) should achieve a critical viscosity (V_crit_) to allow particle-adhesion at the polymer surface while avoiding particle submersion (below V_crit_) or repulsion (above V_crit_) from the surface. A dry-time/particle adhesion study was conducted to identify the “application window” in which particle adhesion to the PDMS binder was optimized. Surface abrasion was conducted with a microfibre fabric surface using a constant weight (0.05 kg) and abrasion rate (0.06 ms^–1^),^49^ and surface robustness was defined as maintaining a static contact of >130°, previously shown as optimal for balancing hydrophobicity and antiviral efficacy (Figure 5 and Figure 7, entry B).

**Figure 8:**
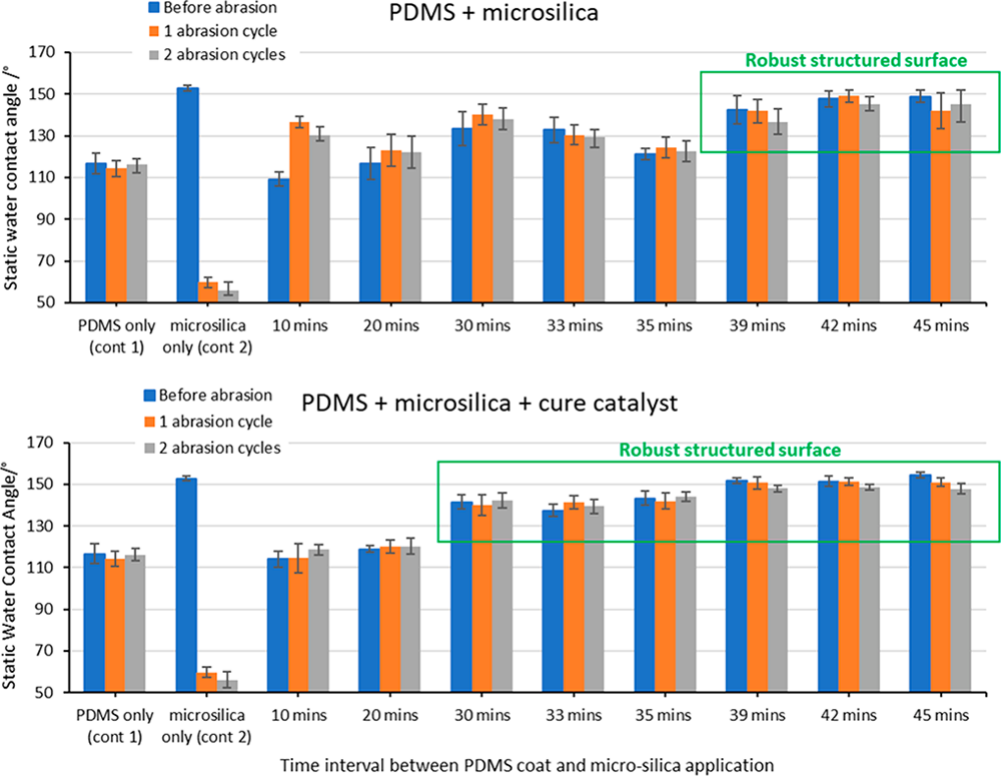
Application window study of particle adhesion to identify the optimal conditions for adhesion of microsilica to a partially cured PDMS adhesive under “normal” (top) and “cure catalyst” accelerated (bottom) conditions.

Figure 8 (top) Entries 1 and 2 shows the control systems of PDMS (entry 1) and microsilica (4.5 μm, C18Si, entry 2) respectively. PDMS (entry 1, column 1 (blue) shows the expected static contact angle of 117° for the PDMS surface, which is robust and does not change upon abrasion (1 or 2 cycles, orange and gray columns respectively). Microsilica coatings applied directly to the substrate shows a static contact angle of ∼150° (Figure 8, entry 2, in-line with previous results, see Figure 2). This value drops to 60° and 56° after 1 and 2 abrasion cycles respectively, which is approximately equal to the static contact angle of untreated glass (52°). These results demonstrate the poor abrasion resistance of microparticle structured surfaces when applied directly to a substrate.

An application window of 10–45 min was studied, for the spray coating of the microsilica suspension over the curing PDMS binder. Figure 8 (top) shows that between 39 and 45 min V_crit_ has been achieved and the microsilica structured surface is present on the surface of the coating. This is characterized by a consistent static contact angle of >130° across the abrasion cycles (Figure 8 (top), Entries 8–10, blue column). These surface show for the first time, no significant loss of hydrophobicity on abrasion testing within one or two cycles (Figure 8 (top), Entries 8–10, orange and gray columns). Further testing revealed that there was no significant loss in hydrophobicity after 10 abrasion cycles (see Supporting Information for details).

A coating system with such a narrow application window is likely to be prone to capricious performance, so expanding it is optimal. The addition cure thermoset PDMS binder (such as Sylgard 184) is commonly cured using a homogeneous platinum-based catalyst (platinum-divinyltetramethyldisiloxane complex), commonly known as Karstedt catalyst,^50^ which is mixed into the liquid (prethermoset) PDMS polymer before application. Higher concentrations of Karstedt catalyst in the PDMS binder would allow faster drying, however this approach would cause other application issues and potentially compromise the material’s performance.^50,51^ It was hypothesized that inclusion of Karstedt catalyst in the microsilica suspension (i.e., the second coat in our process) would accelerate surface cure only (and hardening), while leaving the bulk curing rate unchanged. It was found that this change successfully expanded the application window (Figure 8 (bottom), defined by the time required to achieve a stable static contact angle of >140°). Static contact angles within this new expanded application window (Figure 8 (bottom), highlighted) are consistently higher (Figure 8b, Entries 5–10, blue column) compared to the equivalent time points without additional cure catalyst. Abrasion data (Figure 8 (bottom), Entries 5–10, blue vs orange vs gray columns) also shows improved resistance to mechanical damage (see Supporting Information for details), further highlighting the advantages of this formulation improvement.

**Figure 9:**
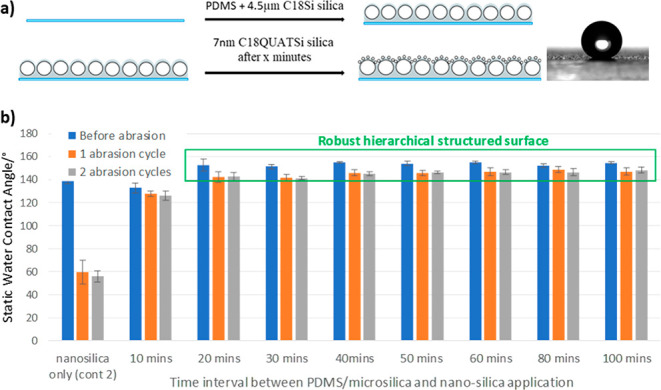
(a) Application process for the creation of robust, nano- on microhierarchical structured coatings. (b) Application window study for the cure catalyst accelerated preparation of robust, nano- on microhierarchical structured coatings.

The final objective was to apply this approach to the hierarchical structured surfaces. Direct application of the technique described above, was not suitable for hierarchical mixtures as the binder would only adhere the microparticle base layer to the substrate. This would leave the functionalized nanoparticles (which occupy the upper layer) attached by relatively weak electrostatic forces to the microstructured base layer. While the topography of the microstructured base layer was clearly important to achieve the overall hydrophobic, antiviral effect, the chemical functionality on the surface of the particle is not critical, as demonstrated previously (Figure 5). Microparticles were therefore suspended within the PDMS binder and sprayed applied to the substrate as thin microstructured base layer. A second spray application of quaternary ammonium functional nanosilica (7 nm, C18QUATSi, 1 wt % in propan-2-ol) was then applied to the partially cured microstructured base layer (Figure 9a). The difference in characteristic static contact angle of PDMS (114–120°), 7 nm (C18QUATSi) nonhierarchical surfaces (135–140°) and 7 nm (C18QUATSi) on 4.5 μm hierarchical surface (140–155°) were used to characterize the resultant surface type, supported by AFM imaging.

An application window and robustness study was conducted on the optimal structured adhesive (PDMS, C18Si microparticle) with a nanoparticle (C18QUATSi) hierarchical upper layer. Catalyst accelerated cure of the nanosurface coating was again effective in promoting robustness and reproducibility of structured surfaces. The “nanoparticle only” coatings (no PDMS binder, Figure 9b, entry 1) show the expected poor mechanical robustness, with complete removal of the nanoparticle layer after a single abrasion cycle. The optimized robust, superhydrophobic hierarchical coatings, in contrast, achieved static contact angles as high as 155°, with excellent abrasion resistance and consistent performance across a wide application window (Figure 9b, entries 2–8, highlighted). Minimal loss of hydrophobicity was observed after multiple abrasion testing (see the Supporting Information for extended test data), highlighting this methodology as producing robust, hierarchical structured surfaces by simple spray application.

**Figure 10:**
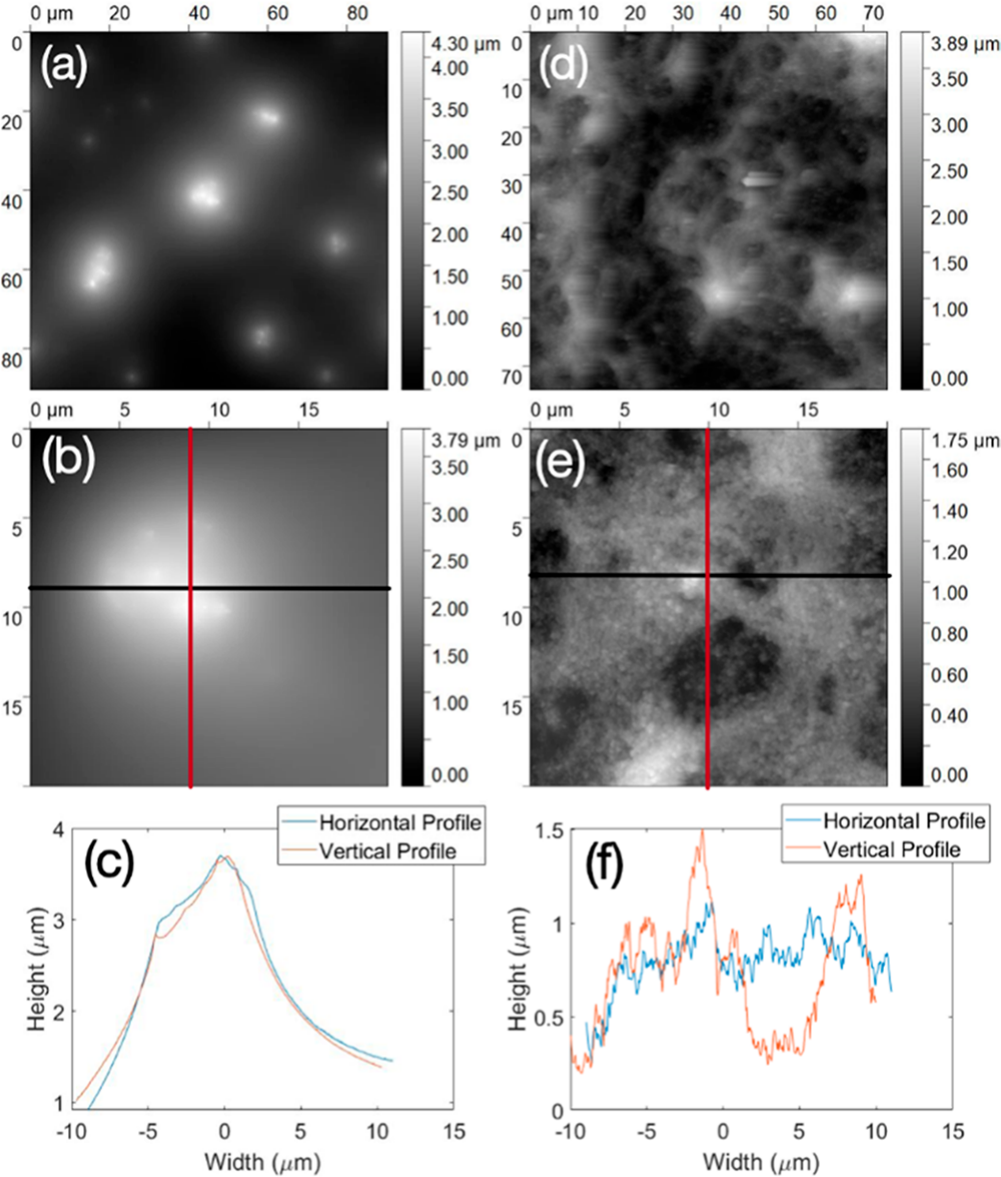
Topographic images acquired using AFM showing the distribution of microparticles, over an area of (a) 90 × 90 μm and (b) 20 × 20 μm on microstructured (PDMS, 4.5 μm, C18Si) base layer. The line scans (c) showing the height of the features on the microstructured base layer. The “nano- on microstructured surfaces” are visible when the hierarchical structured surfaces are scanned over an area of (d) 75 × 75 μm and (e) 20 × 20 μm. The line scan (f) shows resolution of both the micro- and nanosurface features.

Topographic images of both the microstructured PDMS base layer and the hierarchical “nano- on microstructured surfaces” were acquired using AFM tapping mode imaging techniques. The results of these experiments show clear evidence for the hierarchical structure proposed in this report. The samples were imaged over different scan sizes to investigate the single-tier microstructure and the multitier hierarchical structure of the surfaces. Figure 10a shows an image of microstructured base layer (PDMS with microparticle, C18Si) binder sample over an area of 90 × 90 μm, where the presence of 4.5 μm particles over the surface results in the peaks visible in the image. The distribution of microparticles is clearly visible, especially when the scan area is smaller, as shown in Figure 10b. The height of the features can be seen in Figure 10c over the lines labeled in Figure 10b. The same methodology was applied to investigate the hierarchical “nano- on microstructured surfaces” (PDMS with microparticle (C18Si) base layer plus nanoparticle (C18QUATSi) upper layer) as shown in Figure 10d–f. The distribution of 4.5 μm particles results in the larger topographic differences in these images, while the higher-frequency variations (seen in Figure 10f) are due to the presence of C18QUATSi nanoparticles across the surface of the coating. Statistical analysis of different samples is provided in the Supporting Information.

## Conclusions

We report here, for the first time, a versatile method for the creation of self-assembled, multitier structured surfaces that optimize both antiviral and hydrophobic (easy-clean) properties. The methodology exploits the availability of surface-active functional groups and uses the surface micro/nano-structure to control the way the surface coating interacts with molecules and the surrounding environment. This methodology demonstrates significant advantages over single-tier functional structured surfaces, including the ability to overcome droplet-pinning effects. In addition, the methodology presents a simple route to robust structured coatings that maintain both the hierarchical nano- on microsurface features and allows exploitation of surface-active functional groups. This is all achieved using readily available materials and a scalable spray application process using nonspecialized equipment. Given the wide range of surface functionalities that can be appended to hydrophilic silica, which is hence applicable to the hierarchical structured surfaces we describe, this methodology offers significant opportunities to support advances in research in biotechnology, materials engineering, and surface science.

## Supporting Information Available

Supporting video (ZIP)
Spectral data and extended methodologies for synthesis, application, and testing processes (PDF)
