# Supplementary material for: Self-Assembled, Hierarchical Structured Surfaces for Applications in (Super)hydrophobic Antiviral Coatings
Source: Langmuir. 2022 Aug 17;38(34):10632–41. doi: 10.1021/acs.langmuir.2c01579 (PMC9434993; doi:10.1021/acs.langmuir.2c01579)
Supplement: Supplementary file 2 — la2c01579_si_002.pdf [file la2c01579_si_002.pdf]

Supplementary Data

Self-assembled, hierarchical structured surfaces for applications in (super)hydrophobic antiviral coatings.

Frances Dawson[#], Wen C. Yew[#], Bethany Orme[#], Christopher Markwell[#], Rodrigo Ledesma-Aguilar[^], Justin J. Perry[#], Ian M. Shortman[§], Darren Smith[#], Hamdi Torun[#], Gary Wells[^] and Matthew G. Unthank[#*]

AUTHOR ADDRESS

#: Northumbria University, Newcastle upon Tyne NE1 8ST, UK. matthew.unthank@northumbria.ac.uk

§: Defence Science and Technology Laboratory, Porton Down, Salisbury SP4 0JQ, UK.

^: Institute for Multiscale Thermofluids (IMT), School of Engineering, University of Edinburgh, Mayfield Road, EH9 3JL, Scotland, UK.

*: Department of Applied Science, Northumbria University, Newcastle upon Tyne NE1 8ST, UK. matthew.unthank@northumbria.ac.uk

Particle functionalisation method and suspension preparation

OH-functional silica particles (0.75g) were suspended in either toluene (75mL, ANHYD) or ethanol:water (100mL, HYD) using dust extraction system or glovebox suitable for the handling of nano sized materials. The silicas were selected from the table below (Table S1).

Table S1: Silica particle diameters and commercial (Evonik) brand name, used in this study

Silica	Particle diameter /nm
AEROSIL 300	7
AEROSIL 90	20
AEROSIL OX50	40
SIPERNAT 350	4500

Each suspension was treated with silane functionalisation agent (0.75 mmol), selected from n-tetradecyldimethyl(3-trimethoxysilylpropyl)-ammonium chloride, 50% in methanol [C14QUATSi], dimethyloctadecyl[3-(trimethoxysilyl)propyl]ammonium chloride, 60% in methanol [C18QUATSi], trimethoxy(octadecyl)silane [C18Si, control] and *N*-trimethoxysilylpropyl-N,N,N-trimethylammonium chloride, 50% in methanol [QUATSi, control].

Two functionalisation methodologies were used in this research and are described in detail below.

Anhydrous functionalisation method (ANHYD)

OH-functional silica particles (0.75g) were suspended in toluene (75mL, ANHYD) and heated for 4 hours at reflux, under nitrogen with stirring. The suspension was allowed to cool before centrifuge sedimentation (3000 rpm, 10 mins), decanting the solvent from the particles and washing three times with ethanol and once with propan-2-

ol (centrifuging and decanting in-between each washing cycle). The silicas (0.75g) were then suspended in propan-2-ol (75 ml), sonicating with a MSE Soniprep 150 Plus tip sonicator (15 mins, 14 micron amplitude, over ice) to produce a silica suspension for spraying.

Hydrous functionalisation method (HYD)

OH-functional silica particles (0.75g) were suspended in ethanol:water (50:50, 75mL, HYD) and heated for 4 hours at reflux, under nitrogen with stirring. The suspension was allowed to cool before centrifuge sedimentation (3000 rpm, 10 mins), decanting the solvent from the particles and washing three times with ethanol and once with propan-2-ol (centrifuging and decanting in-between each washing cycle). The silicas (0.75g) were then suspended in propan-2-ol (75 ml), sonicating with a MSE Soniprep 150 Plus tip sonicator (15 mins, 14 micron amplitude, over ice) to produce a silica suspension for spraying.

Infrared spectral evidence of particle functionalisation

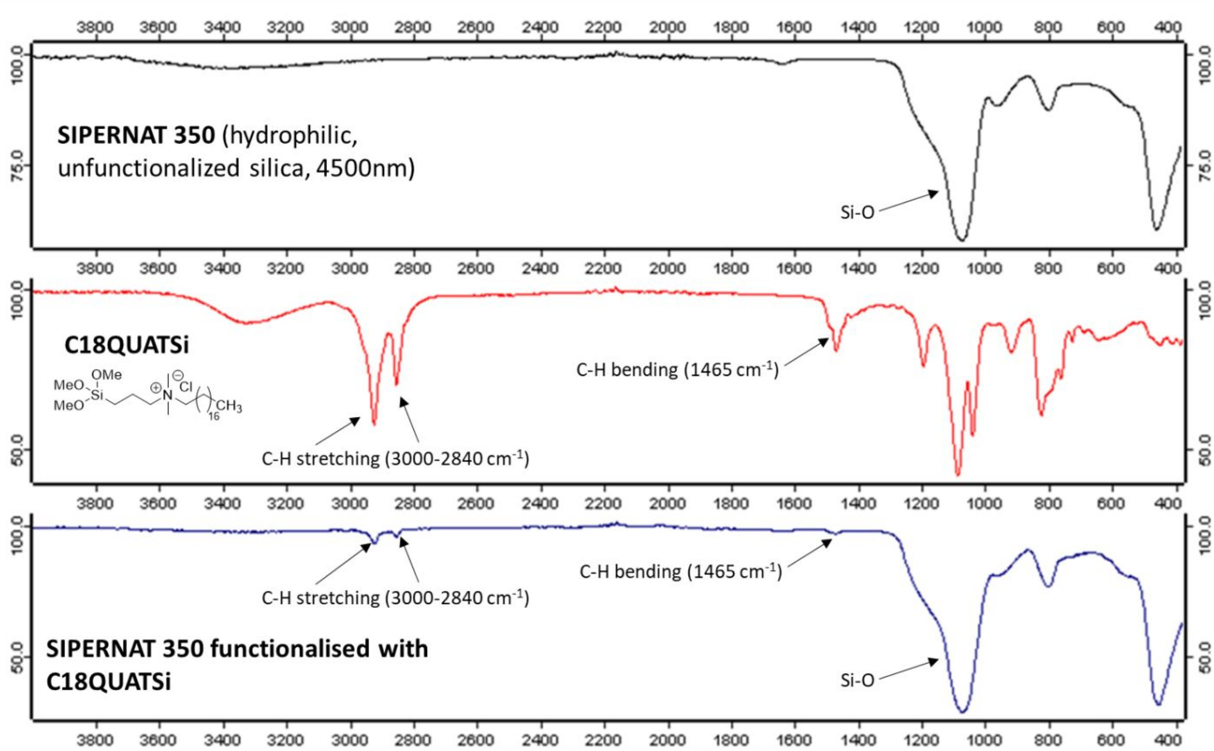

Figure S1: Representative spectra showing the functionalisation of SIPERNAT 350 with C18QUATSi

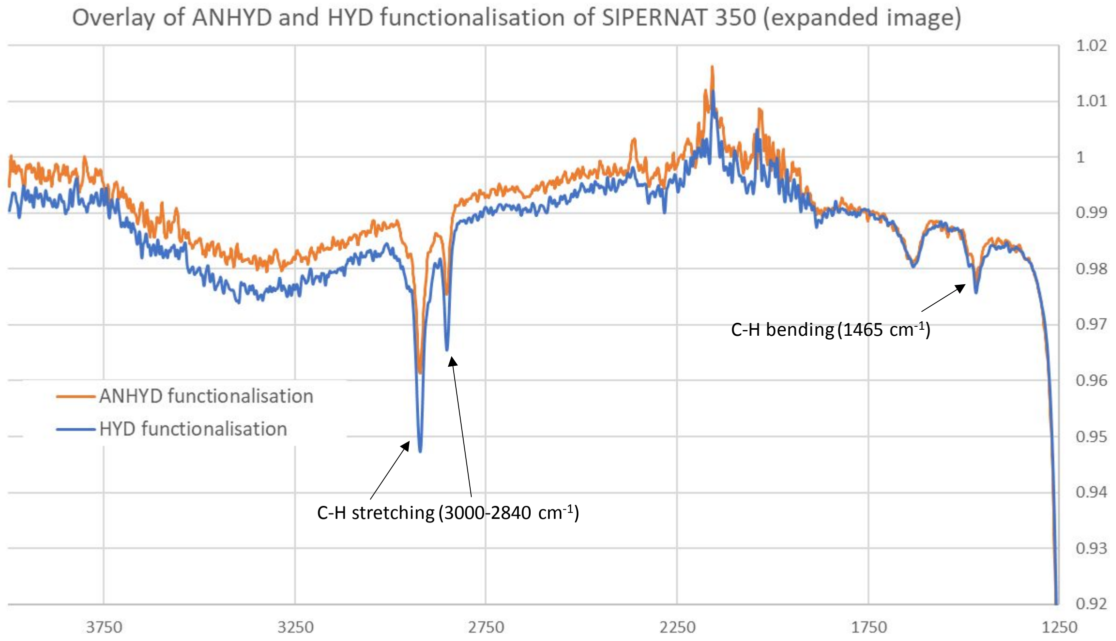

Figure S2: Expanded overlay of ANHYD and HYD functionalisation of SIPERNAT 350, showing the presence of alkyl functionality on the surface of the particles.

Coating application methodology

Single tier-structured coatings

Substrate preparation: Glass microscope coverslips were adhered to microscope slides (3 per slide) with pressure adhesive putty as illustrated in Figure S3.

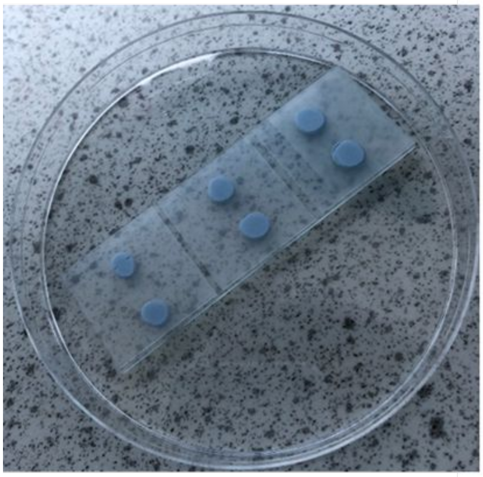

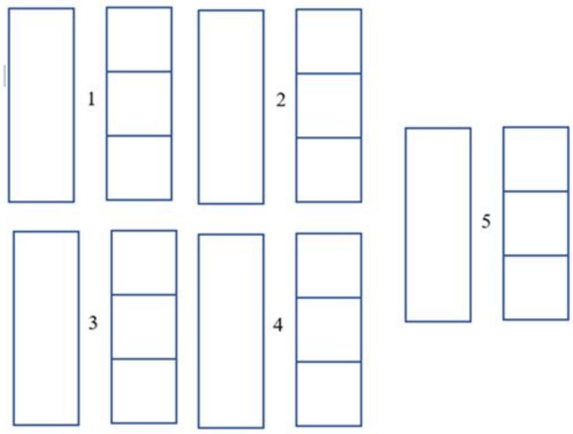

Figure S3: Illustration of substrates spray coated with each functionalised silica sample. For higher throughput antiviral testing, glass coverslips are used as the substrate, with 3 replicas. Top left: 3 coverslips were adhered to and single glass slide with pressure adhesive putty. Top right: For each sample 10 substrates are sprayed with increasing numbers of coats. The sides containing 3 x glass microslide coverslips were used for antiviral testing, the glass microslide slides were used for water contact angle analysis.

Coating application: Nano- and micro-silica suspensions were applied to vertically arranged glass substrates by spray application using a Sparmax spray gun (GP-35) fitted with a hopper and air compressor (Fengda FD-196 Piston Type 186W, 1-2 bar). Initially, 5 mL of propan-2-ol is placed into the spray gun and sprayed onto the glass substrates according to the spraying pattern shown below (Figure S4), at a distance of 15 cm to clean the glass substrate. The silica suspensions were sonicated immediately prior to application using a 60W ultrasonic bath (VGT-1620QTD) for 10 minutes. The silica suspensions (1 wt% in propan-2-ol) were then charged to the

spray gun allowing a volume of 0.5mL per slide, per coat. The suspensions were sprayed according to the spraying pattern illustrated below (Figure S4), allowing 20-30 seconds for the substrates to dry between coats (after which time the coated substrates were visually dry). The coating process was repeated until the desired number of coats was obtained. Once the spray application was complete, the spray gun emptied and cleaned with 5mL of toluene, spraying into tissue paper.

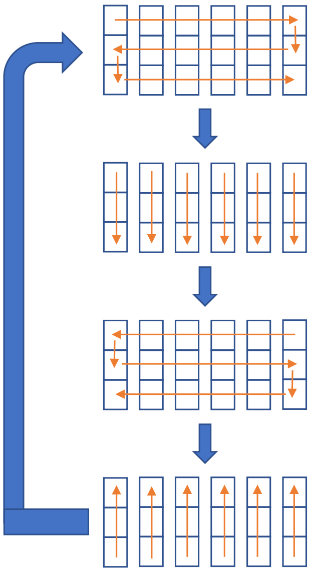

Figure S4: Diagram showing spray coating procedure. The silica suspensions are sprayed on the substrates from a distance of 15 cm, moving the spray gun over the substrates according to the arrows shown above. This cycle of spraying is repeated until the predetermined volume of silica suspension is used up, and this is recorded as a single coat.

Self-assembled hierarchical structured coatings

To prepare the mixed particle size suspensions dispersions, silica solutions of different particle sizes and surface functionalities (1 wt% in propan-2-ol) were combined in a 50:50 volume ratio according to Table S2, and sonicated for 10 minutes to give particle suspension of bimodal particle size distribution. The resulting suspensions were the spray applied onto glass slides via the process described above (Figure S4). In total 4 combinations of hydrophobic/hydrophilic micro and nano silica were tested (Figure S4, Mixture A-C). All silica used were functionalized using the 'anhydrous functionalisation conditions' (refluxed for 4 hours in toluene). For reference, silica functionalized with C18QUATSi is described as 'hydrophobic' and silica functionalized with QUATSi is described as 'hydrophilic'. All silica particle sizes are also labelled in the table and figures below. A non-hierarchical control coating of 40 nm silica (hydrophobic, C18QUATSi) with 40 nm silica (hydrophilic, QUATSi) was also prepared for comparison.

Table S2: Bimodal silica suspension for the creation of self-assembled hierarchical structured coatings.

Mixture	4.5 µm silica	20 nm silica
**A**	C18QUATSi	C18QUATSi
**B**	QUATSi	C18QUATSi

**C**	C18QUATSi	QUATSi
**D**	QUATSi	QUATSi

**Mixture**	**40 nm silica**	**40 nm silica**
**E**	C18QUATSi	QUATSi

Mechanically robust (adhered), single tier-structured coatings: Preparation, Application and Testing

To obtain single-tier robust structured coating a thin film PDMS adhesive binder was used. PDMS (Sylgard 184 base, 1 ml) was diluted with a hexane (19 ml) according to the directions below, to give '*Sylgard solution 1'* . The PDMS and curing agent (Sylgard 184 curing agent) were then combined in a 10:1 ratio by volume, according to the manufacturer's instructions. To facilitate the drying process Karstedt catalyst (Platinum(0)-1,3-divinyl-1,1,3,3-tetramethyldisiloxane complex solution), was also added to the Sylgard formulation according to the directions below. Following application of the PDMS adhesive binder, a suspension of micro-silica (1 wt%) with Karstedt catalyst, in propan-2-ol was applied to deliver a mechanically robust, single tier-structured coating.

Step 1) Adhesive binder preparation and application

0.05 ml of Karstedt catalyst (Platinum(0)-1,3-divinyl-1,1,3,3-tetramethyldisiloxane complex solution), is dissolved in 10 ml hexane to give '*Catalyst Solution A'*

0.05 ml of Karstedt catalyst (Platinum(0)-1,3-divinyl-1,1,3,3-tetramethyldisiloxane complex solution), is dissolved in 10 ml propan-2-ol to give '*Catalyst Solution B'*

1 ml Sylgard 184 base is dissolved in 19 ml hexane to give '*Sylgard solution 1'*.

0.1 ml Sylgard 184 curing agent is added followed by 1ml of '*Catalyst Solution A'*. This mixture is sonicated for 10 minutes then sprayed onto glass slides (2 ml per slide) following the spray application process described above (Figure S4).

Step 2) Functionalised silica preparation and application

A pre-prepared 4.5 µm silica (C18Si functional, AHYDR) suspension in propan-2-ol (1 wt%, 20 ml) was combined with 1 ml of '*Catalyst Solution B' (*as described above) and sonicated for 10 minutes before spraying on top of the PDMS layer at predetermined time intervals, using the standard application process. Samples were cured at ambient temperature for 24 hours.

Step 3) Abrasion testing of the resulting coatings

Surface abrasion was conducted with a microfibre fabric surface at a constant weight (0.05kg) and abrasion rate (0.06 ms^{-1}). The glass slide was placed coating-side down onto a microfibre cleaning cloth and a 50g weight placed on top. The slide is pulled over a 30cm distance over a time of ~5 seconds. Static contact angle were measured before abrasion, and after 1 and 2 abrasion cycles to determine the robustness of the resulting surface. For control silica coatings (without polymeric binder) this process is sufficient to remove the particle layer.

Abrasion Testing of mechanically robust (adhered), single tier-structured coatings

Extended abrasion cycles on robust micro-structured coating whereby micro-silica (4.5 µm, C18Si) is applied to partially cured PDMS adhesive under 'normal' (top) and 'cure catalyst' accelerated (bottom) conditions. 10 repeated abrasion cycles then carried out according to the methodology described above. Figures show no significant loss of hydrophobic behaviour after 10 abrasion cycles.

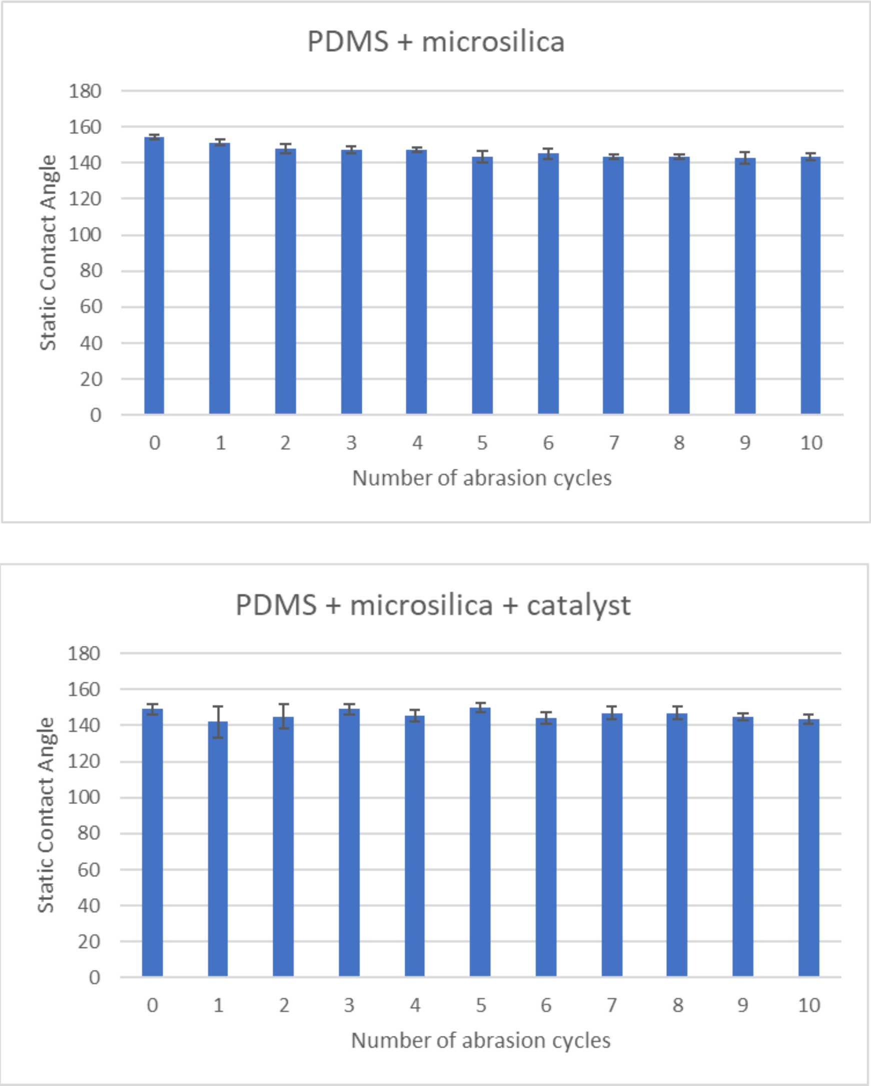

Figure S5: Multiple abrasion cycles demonstrating the robustness of the structured surface

Mechanically robust (adhered), hierarchical structured coatings

To obtain a mechanically robust, hierarchical structured coating with surface active (antiviral) functionality at the surface, the following approach was used.

Step 1) Micro-structured binder preparation and application

'Sylgard solution 1' (10 ml) was combined with the Sylgard curing agent (0.05 ml) and *catalyst solution A* (0.5 ml) and sonicated in an ultrasonic bath for 5 minutes. A pre-prepared 4.5 µm silica (C18Si functional, AHYDR) suspension in propan-2-ol (1 wt%, 10 ml) was added to this solution and sonicated for a further 5 minutes. This mixture was sprayed onto glass microscope slides (x8) and left to dry for 10-60 minutes.

Step 2) Hierarchical functional surface preparation

A suspension of 7nm C18QUATSi (ANHYD) functional nano-particles in propanol-2-ol (1 wt%, 16 ml) was combined with '*Catalyst Solution B'* (1 ml) and sonicated in an ultrasonic bath for 10 minutes. The resulting suspension was then applied to the pre-prepared '*micro-structured PDMS*' substrate, at the pre-determined time intervals of 10-60 minutes, using the standard application procedure. Samples were then cured at ambient temperature for 24 hours.

Step 3) Abrasion testing of the resulting coatings

Surface abrasion was conducted with a microfibre fabric surface at a constant weight (0.05kg) and abrasion rate (0.06 ms^{-1}). The glass slide was placed coating-side down onto a microfibre cleaning cloth and a 50g weight placed on top. The slide is pulled over a 30cm distance over a time of ~5 seconds. Static contact angle were

measured before abrasion, and after 1 and 2 abrasion cycles to determine the robustness of the resulting surface. For control silica coatings (without polymeric binder) this process is sufficient to remove the particle layer.

Abrasion Testing of mechanically robust (adhered), hierarchical structured coatings

Extended abrasion cycles on robust hierarchical structured coating whereby micro-silica (4.5 μm, C18Si) is suspended in the PDMS adhesive and applied to a glass substrate. A nano-silica suspension is then applied to the partially cured PDMS-microsilica basecoat under ‘normal’ (top) and ‘cure catalyst’ accelerated (bottom) conditions. 10 repeated abrasion cycles were then carried out according to the methodology described above. Figures show no significant loss of hydrophobic behaviour after 10 abrasion cycles.

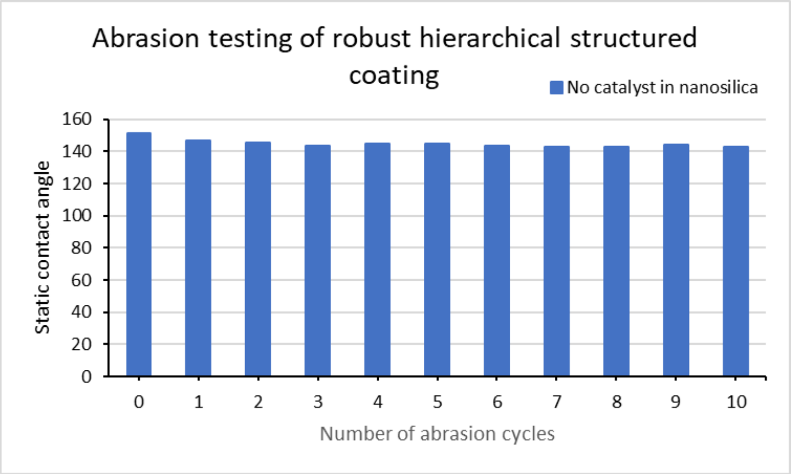

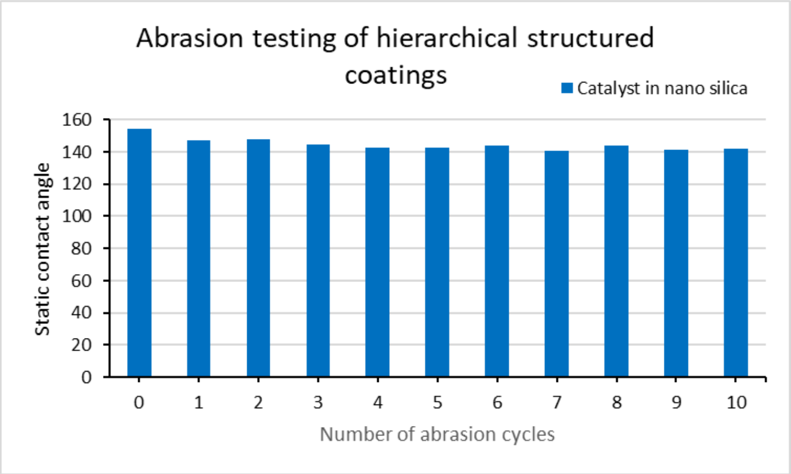

Figure S6: Multiple abrasion cycles demonstrating the robustness of the hierarchical structured surface

Droplet Images examples for structured coatings

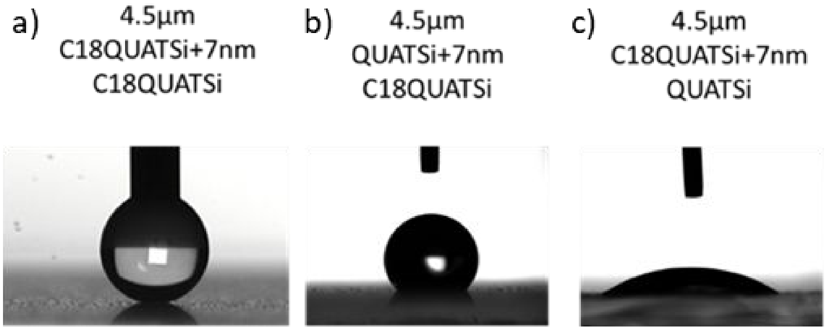

Figure S7: Droplet images for the hierarchical examples described in Figure 5 (Entries 1-3): Shows the static water contact analysis of hierarchical structured surfaces based on 4.5 µm and 7nm functionalized (C18QUATSi and C18Si) silica particles, prepared by single spray application.

Droplet images after spraying micro-silica (4.5 µm, C18Si) onto PDMS binder after different time intervals. Shows the development of a robust hydrophobic surface once sufficient PDMS cure has occurred and micro-particle stay on the surface. The surface cure can be accelerated through inclusion of Pt catalyst in the micro-particle suspension (see main text for details)

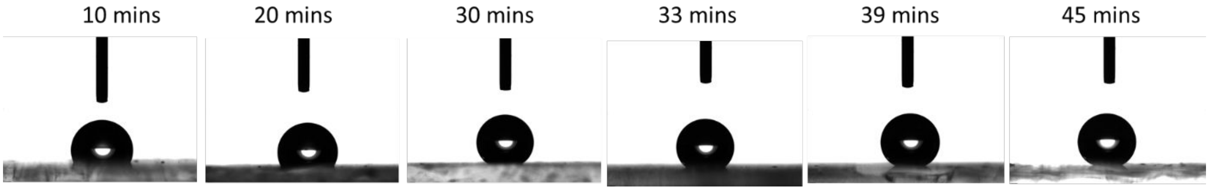

Droplet images showing the impact of reducing PDMS layer thickness when incorporating 4.5 µm silica (C18Si) in the PDMS binder to create robust hierarchical structured surfaces

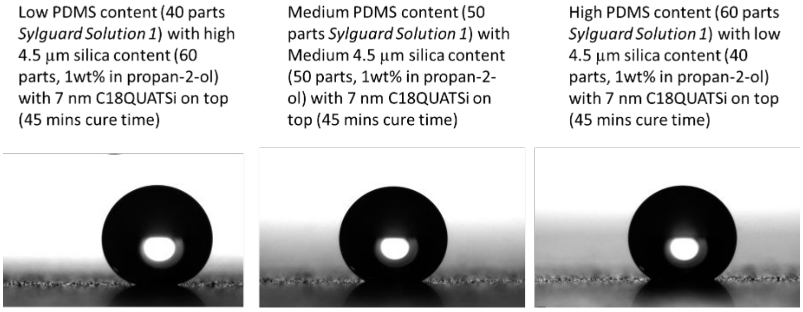

Droplet images show rapid development of a robust superhydrophobic surface from a PDMS-micro silica base layer (4.5 µm, C18Si) with a functional (C18QUATSi) nanostructured surface layer. Prepared following the procedure above for the preparation of '*Mechanically robust (adhered), hierarchical structured coatings*'.

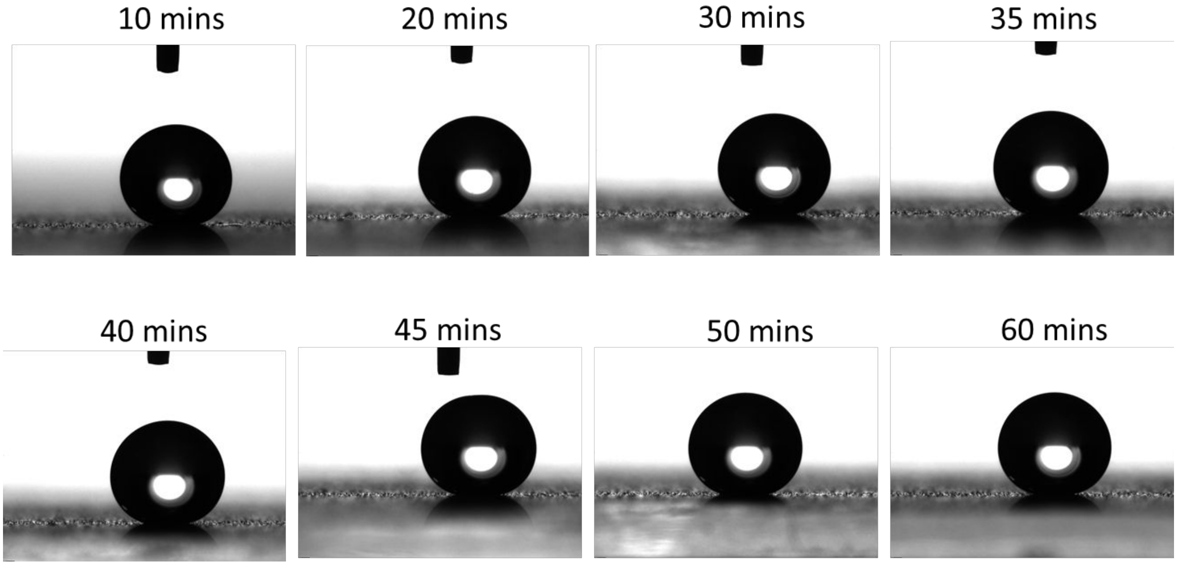

Droplet images show abrasion resistance of hierarchical structured surfaces prepared via the procedure above for the preparation of '*Mechanically robust (adhered), hierarchical structured coatings' (60 minute cure of PDMS-basecoat)*

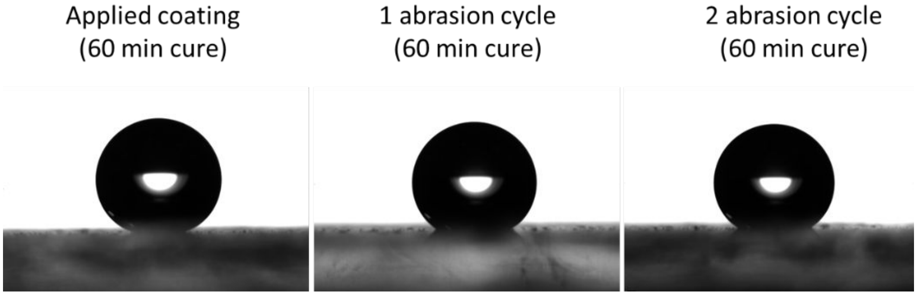

Video of water droplet behaviour on a self-assembled hierarchical structured surface made from C18QUATSi 4.5 micron and C18QUATSi 7nm particles. The video shows the superhydrophobic, water repellent nature of the hierarchical structured surface, where the droplet moves up the micro-syringe needle to avoid the surface.

See supporting video 1

Atomic force microscopy

The topographic images of the coating surface were acquired using a commercial AFM system (Veeco DI3100, Bruker Corporation). Tapping mode imaging techniques were applied using a cantilever with a stiffness of 26 N/m and tip radius of 7 nm (OTESPA, Bruker). The samples were imaged with different scan sizes to investigate the hierarchical structures

AFM images: Self-assembled hierarchical structured coating with 4.5 μm and 20 nm silica particles

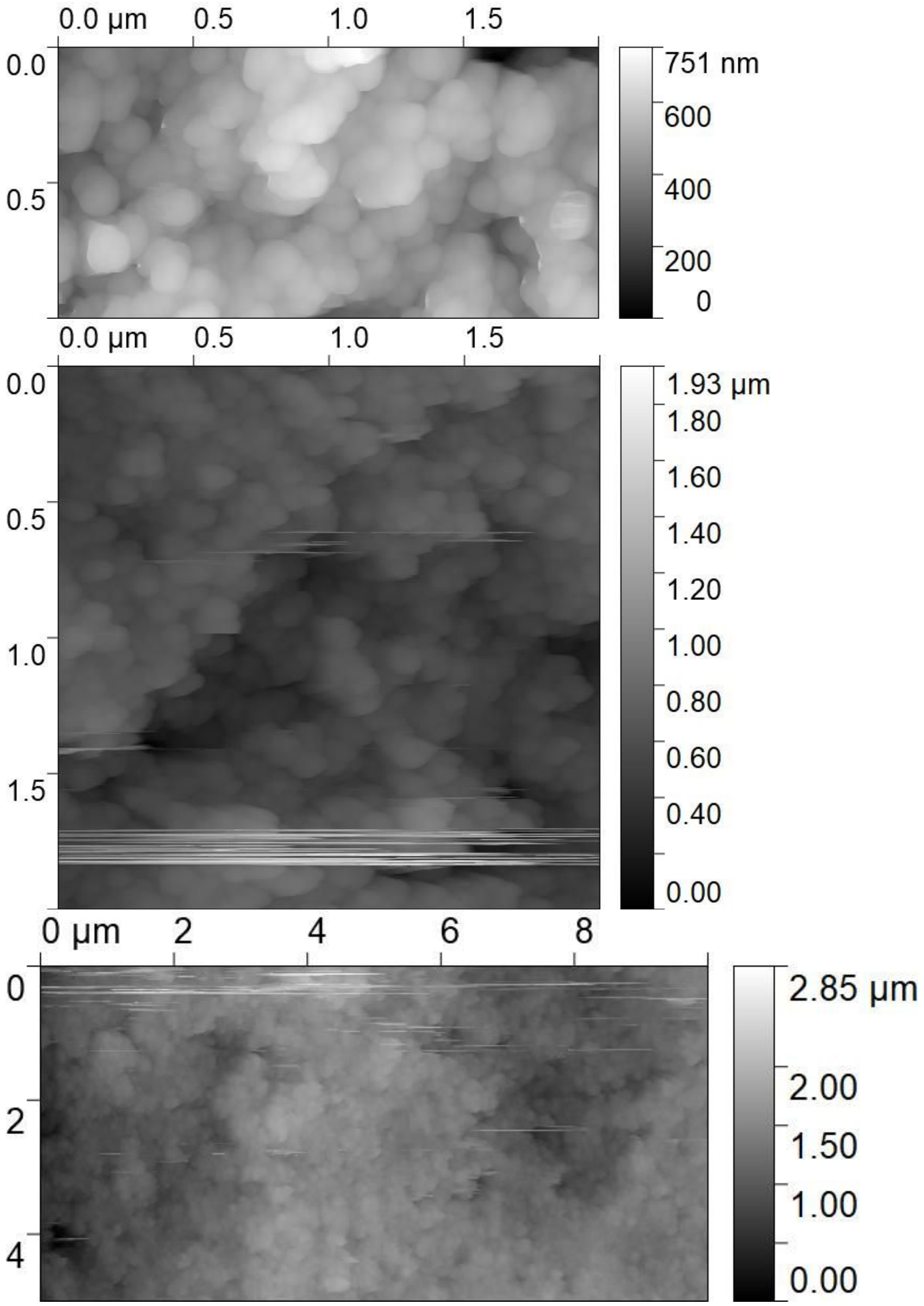

AFM images: Self-assembled hierarchical structured coating with 4.5 µm and 7 nm silica particles

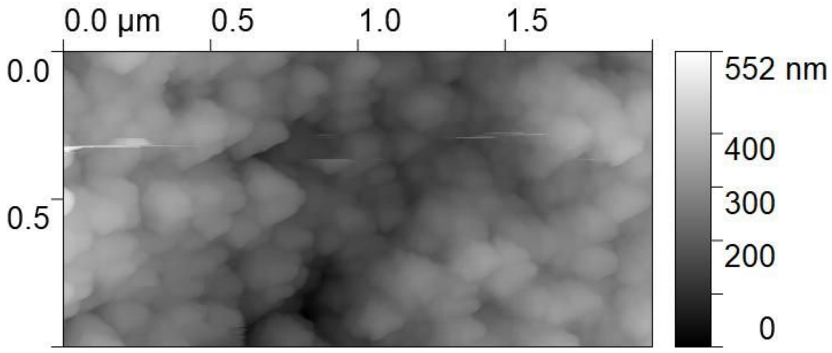

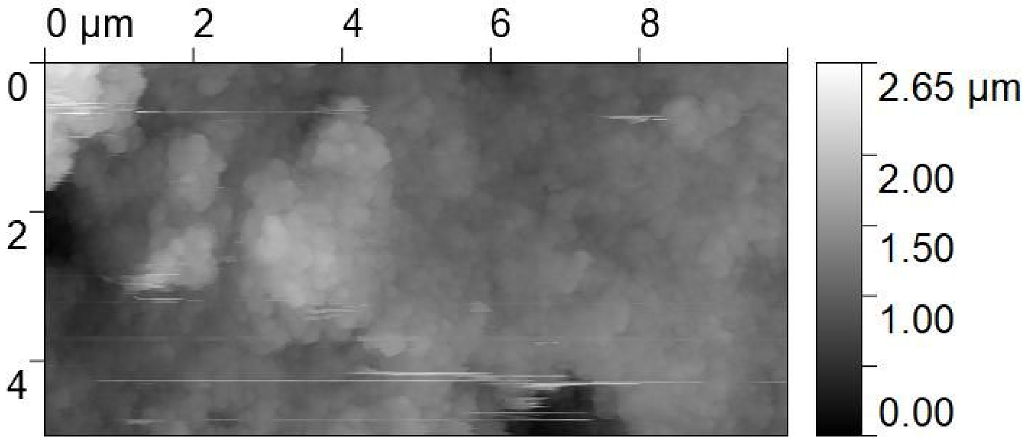

AFM images: The structured surface of Low PDMS content binder (40 parts *Sylguard Solution 1*) with high 4.5 µm silica content (60 parts, 1wt% in propan-2-ol)

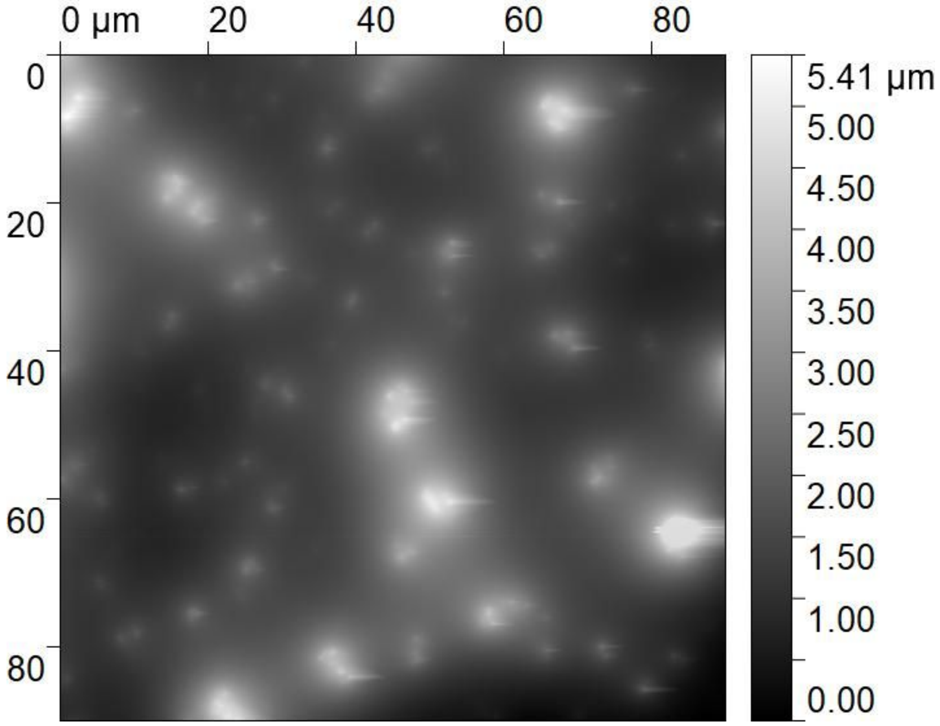

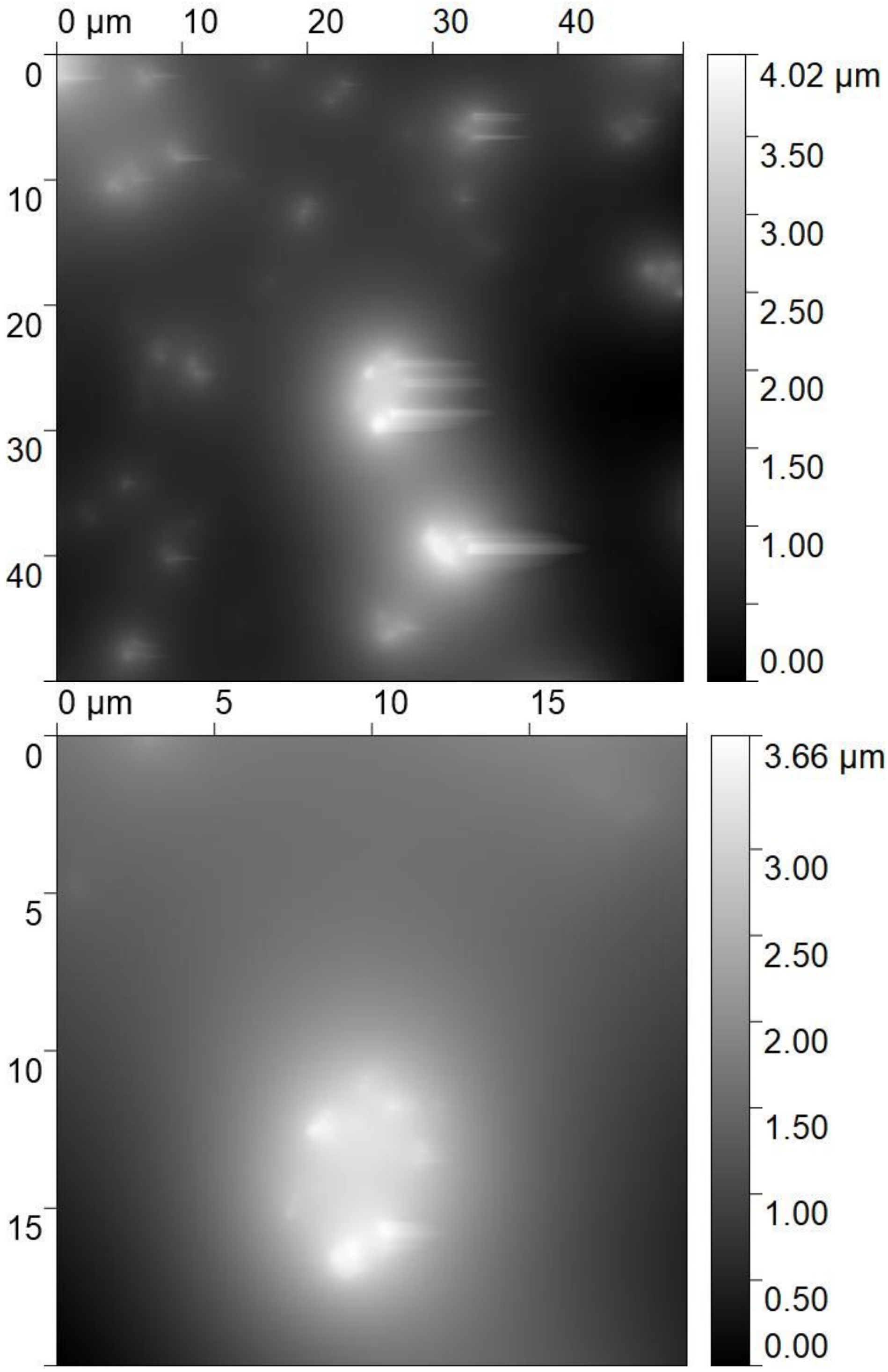
0 µm
10
20
30
40
0
10
20
30
40
4.02 µm
3.50
3.00
2.50
2.00
1.50
1.00
0.00
0 µm
5
10
15
0
5
10
15
3.66 µm
3.00
2.50
2.00
1.50
1.00
0.50
0.00

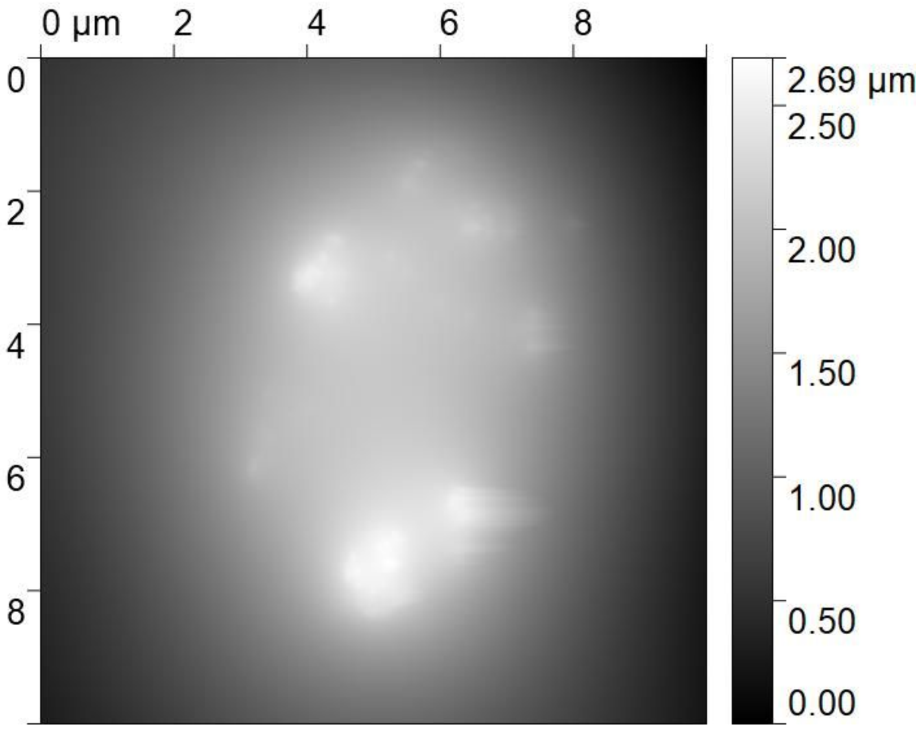
0 μm
2
4
6
8
0
2
4
6
8
2.69 μm
2.50
2.00
1.50
1.00
0.50
0.00

AFM images: The structured surface of medium PDMS content binder (50 parts *Sylguard Solution 1*) with medium 4.5 µm silica content (50 parts, 1wt% in propan-2-ol)

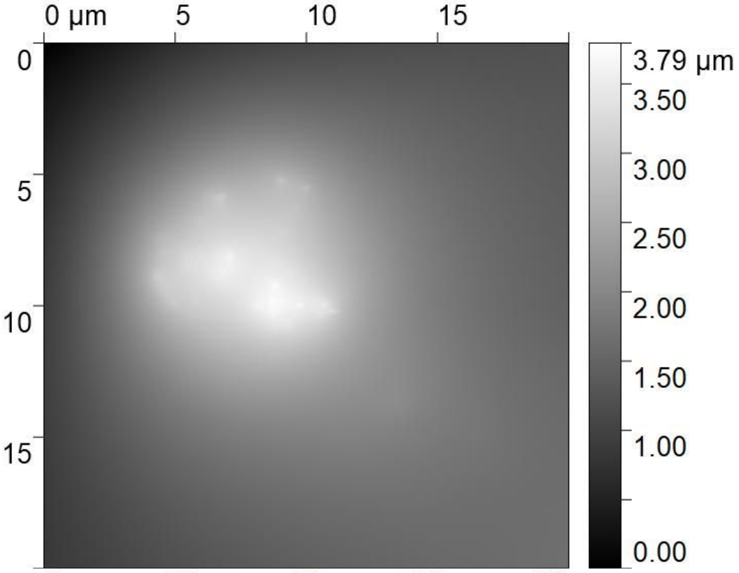

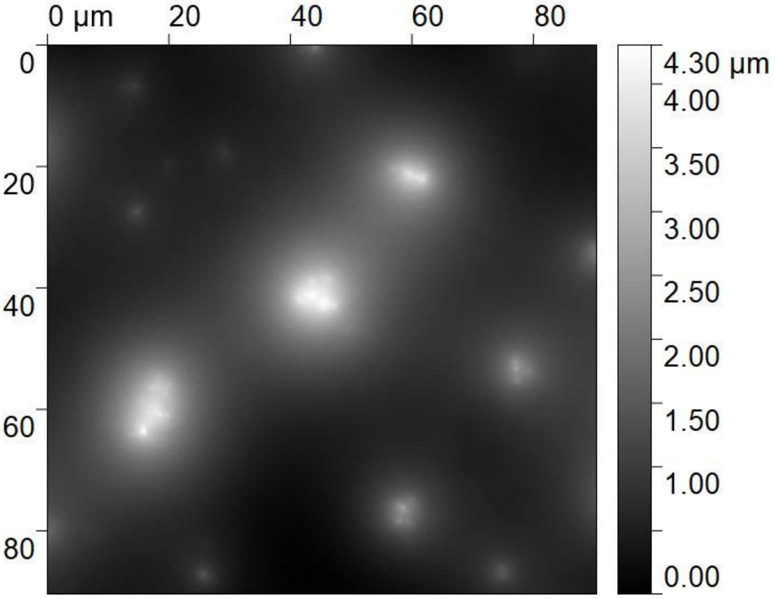

AFM images: The structured surface of high PDMS content binder (60 parts *Sylguard Solution 1*) with low 4.5 µm silica content (40 parts, 1wt% in propan-2-ol)

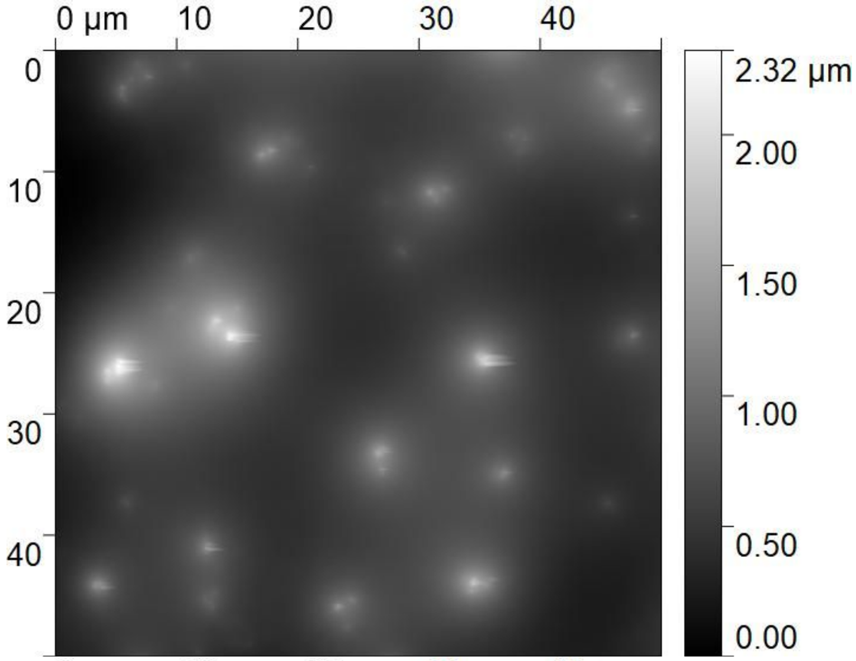

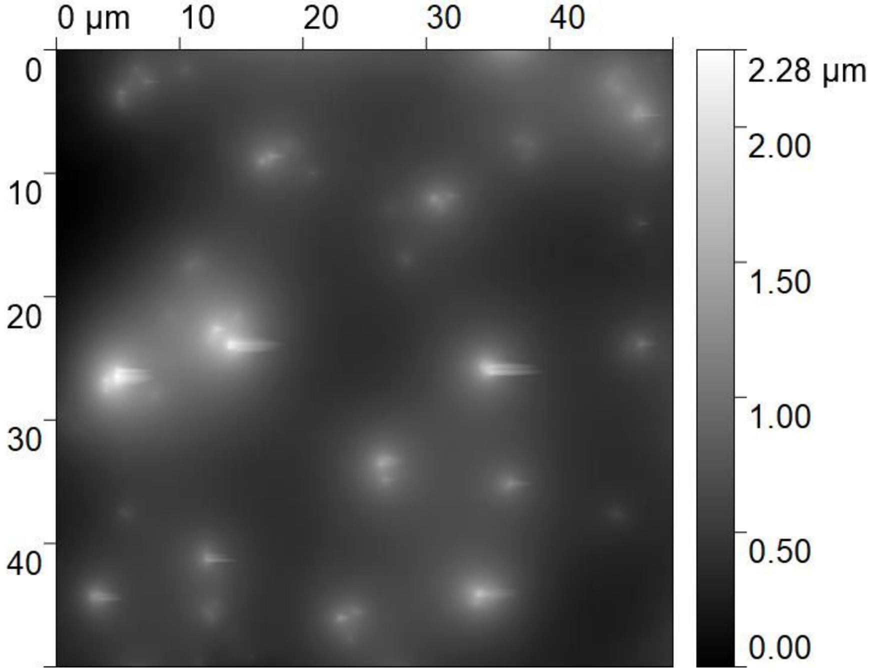

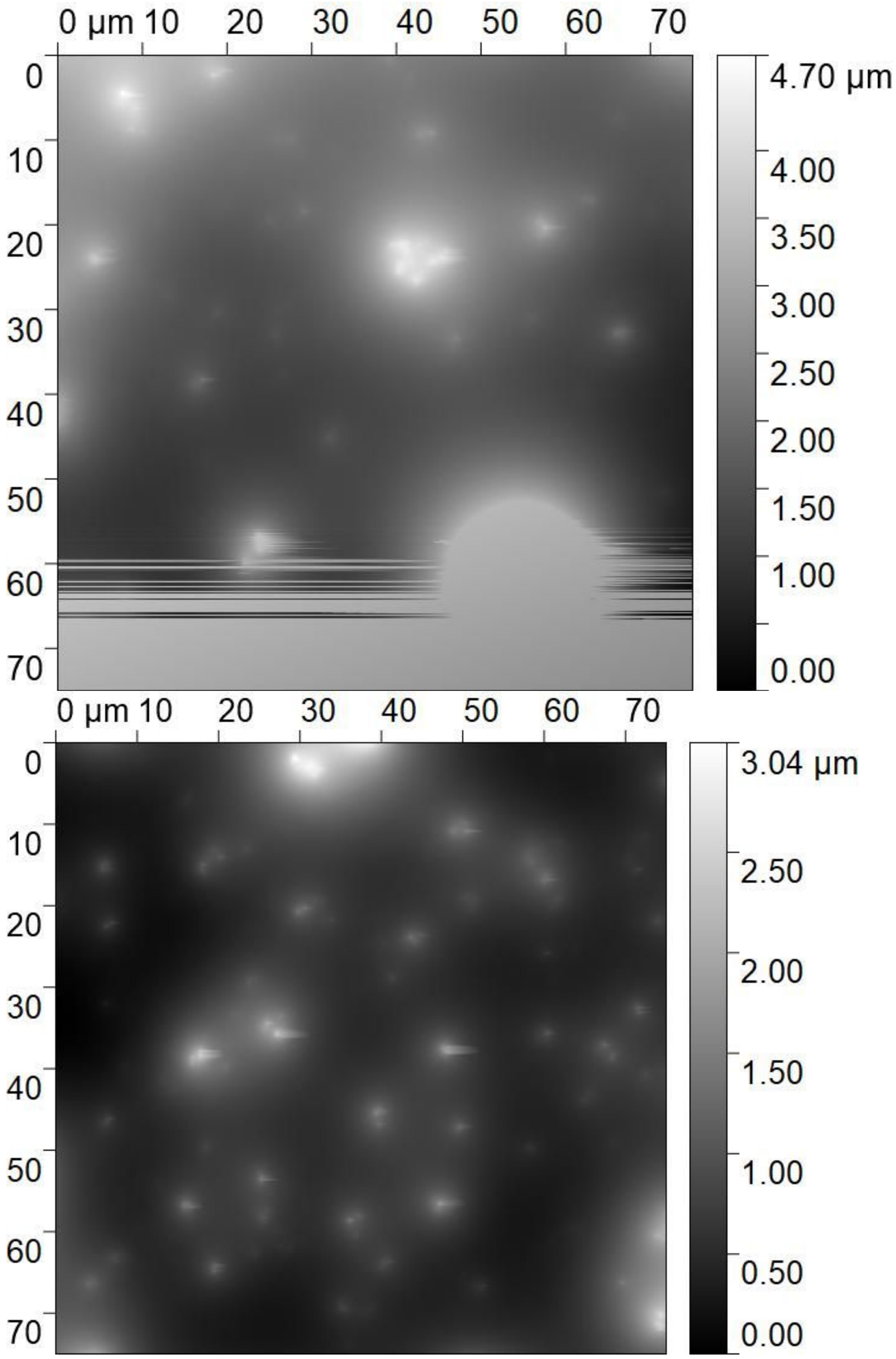
0 µm 10 20 30 40 50 60 70
0 10 20 30 40 50 60 70
4.70 µm
4.00
3.50
3.00
2.50
2.00
1.50
1.00
0.00
0 µm 10 20 30 40 50 60 70
0 10 20 30 40 50 60 70
3.04 µm
2.50
2.00
1.50
1.00
0.50
0.00

AFM images: The structured surface of Low PDMS content binder (40 parts *Sylguard Solution 1*) with high 4.5 μm silica content (60 parts, 1wt% in propan-2-ol) coated with 7 nm functional (C18QUATSi) silica particles to give a mechanically robust hierarchical structured surface. Prepared following the procedure above for the preparation of '*Mechanically robust (adhered), hierarchical structured coatings*'.

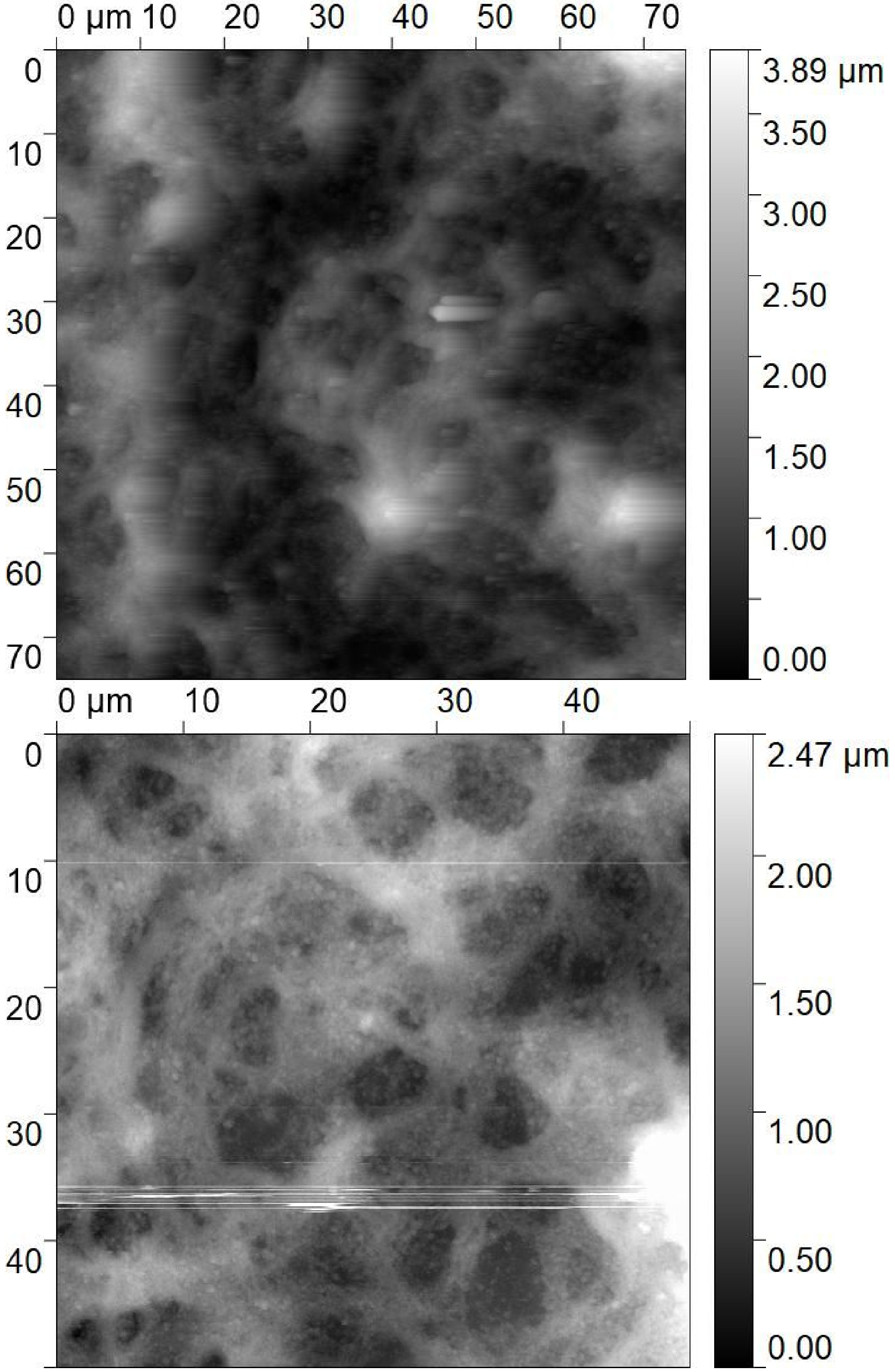

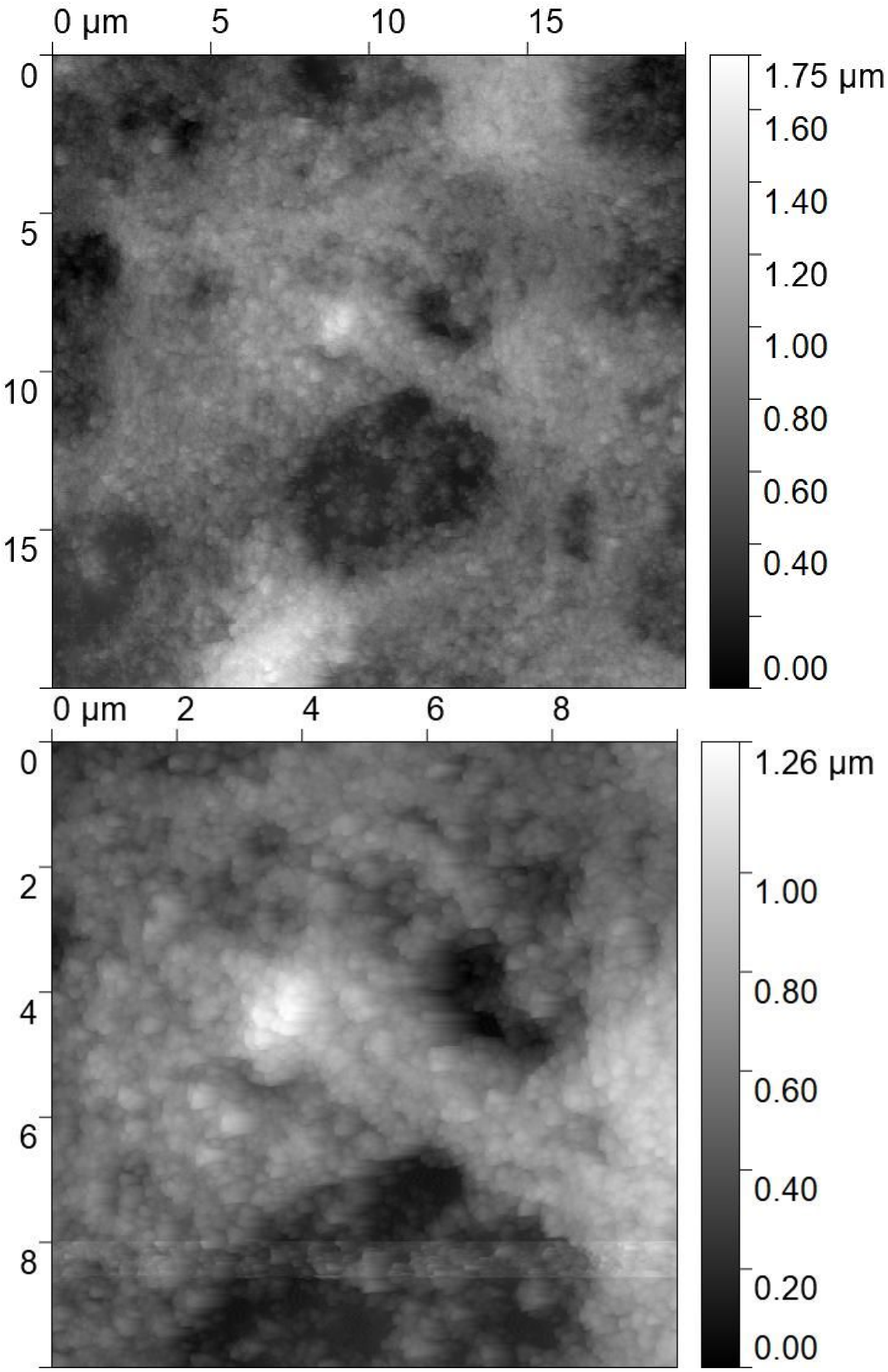
0 µm
5
10
15
0
5
10
15
1.75 µm
1.60
1.40
1.20
1.00
0.80
0.60
0.40
0.00
0 µm
2
4
6
8
0
2
4
6
8
1.26 µm
1.00
0.80
0.60
0.40
0.20
0.00

AFM Supplementary data: Statistical information

Table S3: Statistical information for topographic image shown in Figure 6a

Selected area	512 × 256 pixels
Average value	1.22858 µm
RMS roughness (Sq)	301.023 nm
RMS (grain-wise)	301.023 nm
Mean roughness (Sa)	246.723 nm
Skew (Ssk)	-0.159063
Excess kurtosis	0.275534
Minimum	0 µm
Maximum	2.85018 µm
Median	1.27032 µm
Maximum peak height (Sp)	1.6216 µm
Maximum pit depth (Sv)	1.22858 µm
Maximum height (Sz)	2.85018 µm
Projected area	50 $µm^2$
Surface area	146.141 $µm^2$
Volume	61.4245 $µm^3$
Variation	133.029 $µm^2$
Inclination θ	6.74º
Inclination ϕ	166.64º
Scan line discrepancy	0.0659565

Table S4: Statistical information for topographic image shown in Figure 6b

Selected area	512 × 256 pixels
Average value	459.339 nm
RMS roughness (Sq)	92.681 nm
RMS (grain-wise)	92.681 nm
Mean roughness (Sa)	72.5495 nm
Skew (Ssk)	-0.43645
Excess kurtosis	1.28824
Minimum	0 nm
Maximum	751.112 nm
Median	464.085 nm
Maximum peak height (Sp)	291.774 nm
Maximum pit depth (Sv)	459.339 nm
Maximum height (Sz)	751.112 nm
Projected area	2 $µm^2$
Surface area	4.07037 $µm^2$
Volume	918.729×10^{-21} m^3

Variation	3.30335 µm²
Inclination θ	6.9º
Inclination ϕ	117.75º
Scan line discrepancy	17.0841×10⁻³

Table S5: Statistical information for topographic image shown in Figure 11a

Selected area	512 × 512 pixels
Average value	0.9251 µm
RMS roughness (Sq)	588.661 nm
RMS (grain-wise)	588.661 nm
Mean roughness (Sa)	418.054 nm
Skew (Ssk)	1.93576
Excess kurtosis	5.14237
Minimum	0 µm
Maximum	4.3013 µm
Median	0.7661 µm
Maximum peak height (Sp)	3.3762 µm
Maximum pit depth (Sv)	0.9251 µm
Maximum height (Sz)	4.3013 µm
Projected area	8171.17 µm²
Surface area	8300.64 µm²
Surface slope (Sdq)	0.195916
Volume	7559.14 µm³
Variation	1340.72 µm²
Inclination θ	0.06º
Inclination ϕ	-71.94º
Scan line discrepancy	15.9139×10⁻³

Table S6: Statistical information for topographic image shown in Figure 11b

Selected area	512 × 512 pixels
Average value	1.73088 µm
RMS roughness (Sq)	620.079 nm
RMS (grain-wise)	620.079 nm
Mean roughness (Sa)	459.862 nm
Skew (Ssk)	0.798624
Excess kurtosis	0.81348
Minimum	0 µm
Maximum	3.79096 µm
Median	1.6232 µm
Maximum peak height (Sp)	2.06007 µm

Maximum pit depth (Sv)	1.73088 µm
Maximum height (Sz)	3.79096 µm
Projected area	400 µm²
Surface area	418.209 µm²
Surface slope (Sdq)	0.329641
Volume	692.353 µm³
Variation	115.154 µm²
Inclination θ	2.39º
Inclination ϕ	147.13º
Scan line discrepancy	2.71412×10^{-3}

Table S7: Statistical information for topographic image shown in Figure 11d

Selected area	512 × 512 pixels
Average value	1.06047 µm
RMS roughness (Sq)	583.378 nm
RMS (grain-wise)	583.378 nm
Mean roughness (Sa)	457.869 nm
Skew (Ssk)	0.91675
Excess kurtosis	1.08732
Minimum	0 µm
Maximum	3.89215 µm
Median	0.98123 µm
Maximum peak height (Sp)	2.83168 µm
Maximum pit depth (Sv)	1.06047 µm
Maximum height (Sz)	3.89215 µm
Projected area	5625 µm²
Surface area	6155.38 µm²
Volume	5965.15 µm³
Variation	2291.59 µm²
Inclination θ	0.61º
Inclination ϕ	-61.79º
Scan line discrepancy	0.0324002

Table S8: Statistical information for topographic image shown in Figure 11e

Selected area	512 × 512 pixels
Average value	0.67627 µm
RMS roughness (Sq)	243.785 nm
RMS (grain-wise)	243.785 nm
Mean roughness (Sa)	190.484 nm

Skew (Ssk)	0.294947
Excess kurtosis	0.497025
Minimum	0 µm
Maximum	1.74915 µm
Median	0.68661 µm
Maximum peak height (Sp)	1.07287 µm
Maximum pit depth (Sv)	0.67627 µm
Maximum height (Sz)	1.74915 µm
Projected area	400 μm^2
Surface area	573.038 μm^2
Volume	270.51 μm^3
Variation	400.065 μm^2
Inclination θ	1.46º
Inclination ϕ	48.89º
Scan line discrepancy	31.3632×10^{-3}
